# Fabric defect detection using fine-tuned Yolo-12

**DOI:** 10.1371/journal.pone.0353550

**Published:** 2026-07-22

**Authors:** Waqar Ahmad, Rehan Ashraf, Toqeer Mahmood, Tanveer Hussain, Muhammad Haris Abid

**Affiliations:** Department of Computer Science, National Textile University, Faisalabad, Punjab, Pakistan; King Mongkut’s University of Technology North Bangkok, THAILAND

## Abstract

Defect identification is critical for ensuring the reliability and price of fabrics. Defective fabrics result in significant waste and losses. Automatic defect identification using the use of deep learning is a faster and more efficient way to analyze fabric quality, replacing human inspection. Furthermore, both plain and printed textiles are produced concurrently in enterprises; hence, one design should be effective in identifying faults in both types of fabric. As a result, a strong deep learning algorithm must be trained to detect defects within fabric datasets produced during manufacturing with excellent performance and cheap computing costs. This study incorporates a local dataset collected from Chenab Textiles and validated with three publicly available datasets, such as Tildav2, DPFD-DET, and ZJU-Leaper. The experiment provides a comprehensive and diversified range of defective images. To identify textile defects successfully, the suggested approach, universal and optimized YOLOv12, named universal defect detect network (UniDefectNet-Omni) for robust identification of a wide spectrum of fabric defects with multi and diverse types of fabric using YOLOv12 by fine-tuning and optimizing training, integrating high determination feature learning, heterogeneous defect representation, and adaptive augmentation. As a consequence, UniDefectNet-Omni is a lightweight, computationally efficient, and robust framework across varied fabrics.The Chenab textile dataset mean Average Precision (mAP) is 85.1%, precision is 84.5%, and recall is 81.7% over seven separate fabric defect categories. The proposed fine-tuned YOLOv12 outperformed on validated datasets, such as the TILDAv2 dataset, having a mean Average Precision (mAP) score 86.7%, precision about 83.7%, in addition recall about 83.6% over four separate fabric defect categories. DPFD-DET with a mean Average Precision (mAP) score 93.6%, precision is 92.2%, and recall is 87.8% over four separate fabric defect categories. ZJU-Leaper with groups 1, 2, 3, and 4 having a mean Average Precision (mAP) score 93%, precision about 78.1%, in addition recall about 90.3% over twelve separate fabric defect categories.

## 1 Introduction

The clothing sector is among the world’s foundational and significant industries, driving economic growth, job creation, and technological advances. Textiles are especially important in nations like Pakistan, where they account for a sizable portion of exports and industrial production. Textile production, through weaving or spinning with dyeing as well as finishing, is actually a complicated process that requires high levels of precision and reliability [1]. Textile quality assurance is a vital stage throughout the manufacturing process, with substantial implications for product quality, costs, economic efficiency, and the competitive advantage of organizations. Fabric defect identification within the textile industry is a difficult task. Defects in textiles not only influence the look, durability, and longevity of items, but they may also cut pricing by 45% to 65%, emphasizing the crucial need for accurate defect identification. As a result, fast and precise detection of fabric problems is critical for assuring product quality. Accurate detection may prevent faulty items from accessing the market. Whereas identifying possible issues early allows for immediate remedial action to mitigate negative effects on corporate image and consumer satisfaction [2]. Fabric becomes wasted mainly as a result of numerous production defects. This causes financial losses for textile producers throughout Pakistan. Machines’ failure is among the most common sources of these faults. An inspection is necessary to ensure that the manufactured cloth meets market requirements. The inspection requires several competent people. The process is physically demanding and may lead to mistakes due to weariness. Hence, a computerized methodology is necessary for this activity. Deep learning-based identification approaches have gained popularity due to advancements in hardware technology [1].

The majority of fabric defect detection research depends on well-positioned photographs obtained under controlled settings. Real-time manufacturing presents unique issues for dataset collections. Images neither have high quality or perfectly positioned. Modifications in lighting intensity, disruption, and blurriness may drastically change visual perception. Furthermore, the literature addressing this subject may be divided into two major categories: faults in plain textiles and defects within printed fabrics. Textured textiles can be classified further into regular as well as irregular prints [3]. The diversity of printing makes it challenging to distinguish between the pattern and the defect. As a result, various approaches must be followed in each circumstance. There is minimal research on models that can detect defects in plain, typical, and irregularly designed textiles [4]. This paper proposes a universal and optimized YOLOv12 named universal defect detect network (UniDefectNet-Omni). UniDefectNet-Omni is designed for robust identification of a wide spectrum of fabric defects with multiple and diverse types of fabric. UniDefectNet-Omni is trained with the Chenab textile dataset and validated with heterogeneous datasets. The UniDefectNet-Omni is designed for both printed and plain fabrics, including grayscale and color variations. UniDefectNet-Omni can detect complex and fine-grained defects such as stains, knots, broken ends, thread errors, holes, and structural inconsistencies. The proposed UniDefectNet-Omni is considered reliable, optimized, and robust for varying production inspections. In this study,

- Indigenous datasets regarding Chenab Textile, Tildav2, DPFD, and ZJU-Leaper were used, including printed with plain fabric photographs obtained under various production settings.
- The object detection framework UniDefectNet-Omni, being computationally quicker and using fewer resources, was trained.
- YOLOv12 was fine-tuned through a thorough hyperparameter tuning and training workflow, which increases resilience against fluctuations in defect form, size, texture, and illumination conditions.

The remaining articles are grouped as follows: Section two provides an overview of the published research on this issue. Section 3 discusses the models along with collections. Section 4 outlines the findings, whereas Section 5 concludes the work and recommends areas for refinement.

## 2 Related work

Fabric defect identification has received a great deal of attention in studies. This field included extensive research throughout history. Fabric identification techniques fall into four categories: statistical, spectral, approach, and deep learning. Statistics algorithms include tools such as the co-occurrence matrix and structure. Manual visual examination is the primary approach for detecting fabric defects, but it is ineffective and has an optimal speed of just twelve meters per minute. According to studies, manually fault identification is accurate between 60% and 75% about the time. Furthermore, manual visual inspection was prone to inspector tiredness, experience, including subjective judgment, which might impair the precision of detection findings [5,6]. The defect is identified by contrasting the gray levels of pixels to those around them. The detection outcomes for such techniques are determined by the extent of the windows. Furthermore, tiny defects are hard to discover using such approaches. Spectral-based techniques transform the investigated image towards the regularity domain. To discover faults, calculate the difference between spectral coefficients. This contains the Fourier transformation technique, the Wavelet Transformed method, and the Gabor Transformed method. Choosing the right filter banks is crucial for the efficacy of these approaches, which also need complicated computations. Model-based approaches characterize textural properties of non-defective materials using various measurement methods. Defects are discovered by comparing test images to the established typical texture model [7,8]. Such models, including statistical models, have high computational complexity and are generally unsuccessful at spotting minor defects. The Auto Regression Approach is a sample. Convolutional neural systems excel at expressing features in computer vision problems. Large convolutional networks require streamlined storage and processing. The research on using neural networks to identify fabric faults is separated into two categories: printed textiles and plain materials.

An inflatable neural network utilized YOLO, was proposed [9] to detect fabric problems. This project aimed to lower computing costs and apply the model to practical uses on embedded systems. Deep CNNs have become more accurate; however, this has led to higher computational and storage demands. This may limit their application in low-resource situations like mobile phones and embedded gadgets. The research offers short convolution levels (1 × among 3 × 3) to decrease dimensionality and merge information. Multi-scale feature collection was employed to improve the model’s detection of different fault levels. K-means clustering was employed to determine the optimal dimensions for anchor frames for YOLO identification in the textile defect image collection. A textile image benchmark collection with 3000 images and five classifications yielded a reliability of 97.2%. An unsupervised learning strategy was developed to detect fabric defects, with an emphasis on collecting faulty samples [10]. Adding annotations to datasets manually becomes time-consuming and costly. This research proposes a sophisticated convolutional generative adversarial network.Researchers utilized a normal DCGAN containing a novel encoder element to recreate the query picture without errors. The encoder produced a residual mapping by subtracting the reconstructed original images. This map identifies possible defect areas. The model generates the possibility map with each pixel within the picture, indicating the probability of faults at that location. The probability and residual maps were combined to create a fusion mapping. The map organizes gray levels within defect-free zones and shows deviations in defective spots. The model had an FNR equal to 12.09, a predictive value of about 51.62%, using an FPR equal to 49.91% for TILDA textile pattern and neighborhood samples. Still, the model produced noisy partitioning. Another multitasking fusion module alongside a focused method was developed to identify fabric defects [2]. The system of attention causes networks to focus on defects. Multi-task fusion improves the proposed architectural categorization through feature concatenation. This fusion component combines attention mapping with classification branches to improve classification results, especially for tiny errors. According to the study, the concept is feasible in real-time commercial cases. The suggested model had an F1 index of about 0.987, a recall of 0.994, and an accuracy of 0.98 within the AITEX collection. Although it was just used with basic materials.

A deeply convolutional neural network was suggested to identify printed fabric problems based on real-time visual data from industries [11]. The fault classifications employed were color recognition and print inconsistencies. Experiments were conducted to determine the optimal hyperparameters using a basic CNN model. The rates of learning (0.0003), the volume of batches (16), and the regularization ratio (λ = 0.001), especially activating the ReLU, have been finalized. The research used VGG-16 through VGG-19 architectures on the collection. The dataset included self-collected imagery printed with two classes: color patches, particularly misprints. VGG16 outperformed all three designs, achieving a recall of about 0.71, a precision of nearly 0.70, and a precision of 72%. TILDA RGBAAM, together with IMAGE PYRAMID are suggested to identify faults in printed textiles with regular patterns [12]. The first stage was calculating the minimum printing duration for the cloth using RGBAAM. The minimal time was utilized as a foundation to create a Gaussian pyramid using both the defective and template images. The template, while a faulty picture, was compared using an identical measuring approach. Finally, faults in printed cloth were identified through the Laplacian pyramidal repair approach. According to the study, the suggested model accurately determines the periodic element for printing as well as the fault site. Complex patterns take longer for the framework to perform. Zhang et al. proposed an economical MobileNetV2-SSDLite enabling cloud-edge computation [13]. The model uses channel concentration and focused loss to identify minor defects and maintain a balance across faulty and normal data. Experiments were done using four distinct datasets. The suggested technique attained accuracy scores of 84.39 with CF, 93.05 with GF, 71.18% with BPF, and 95.5% with the DRF dataset. Zhao et al. upgraded the faster RCNN, which was added for identifying defects [14].

This approach aims to improve accuracy and convergence when identifying small faults. The quicker RCNN was modified to use a ResNet50 basis rather than VGG16. This resolved the issue of the gradients disappearing due to the Res-Net’s increased depth with residual connections. Another FPN was added for linking low- and high-level characteristics, improving the accuracy of detecting tiny faults. ROI pooling has been used rather than ROI matching. The benefit was that quantization had been reduced, and therefore, nuances were not overlooked. Furthermore, transfer learning has been employed to reduce the duration of training. The suggested architecture was evaluated with self-collected fiber samples. The defects involved ribbon yarn, damaged yarn, holes, and stains. This model achieved a mAp scoring of 94.73% based on the provided dataset. Although the architecture had not been implemented in production. The VGG system was used to classify five distinct fabric faults [15,16]. The study developed an initial processing filter to remove nonlinear mixed noises from pictures and presented a deeply trained CNN framework to detect faults. The research consists of two parts. In the beginning stage, a pseudo-convolutional neural network (P-CNN) is utilized for picture preprocessing. The CNN network has been constructed containing three layers, similar to standard convolutional networks [17,18]. The earliest feature extraction stages employ weight-initiated adaptive window filtering. These filter parameters are initialized through a probability pattern of noise. This PCNN effectively rejects impulsive noise in pictures. Stage 2 includes a CNN to categorize and find problems. The self-collected collection yielded 93.92% accuracy along with 92.51% specificity. Still, the algorithm failed to accurately detect printed textiles with bands versus real-time ordinary fabric samples containing noise.

A dual sparse low-rank decomposing approach was developed to detect faults in printed textiles with irregular and complicated patterns [19]. The suggested model consists of three successive steps. Initially, previous knowledge was retrieved from both kinds of data: templates and defects. The template was previously produced from sparse materials and used as a printable template. The incorrect prior was identified by comparing the faulty printed fabric structure to the template fabric graph. The backdrop and print were separated using a double-dense low-rank decomposition. To segment faults, prominence mAp proved binaries using an optimum threshold segmentation approach. This method helped identify and see faults more clearly. Using a self-collected collection of 98 cloth designs, the model achieved a TPR of about 89.29%, with an FPR of about 0.85. Although the model is not very robust. SDANet (in Siamese FPN) had been scheduled to identify faults in printed textiles [20]. The Siamese featuring pyramid network has been employed to extract multi-scale attributes from both inputs alongside standard/template images. An attention component was developed to identify differences between source and template characteristics. A self-calibration device was developed to reduce positioning errors between the standard along input images. This research employed two well-known datasets: Tianchi Fabric and Tianchi Tile Defects Identification. The model achieved a mAp of about 47.1, with an accuracy of about 83.3%. The approach has the issue of requiring template photos for every design to detect faults. Quite recently, Li et al. trained a strong model using a fabric collection that included both printed with plain materials [21]. The researchers used a cascade R-CNN using a self-collected collection with 19 distinct backdrops and 9 categories, including stains, gaps, wrinkles, and thread ends. Additional strategies were used to increase the model’s precision. The “block identification and detection package merging algorithm” detects tiny and medium-sized faults in high-resolution pictures. To train, big, high-resolution photos were broken into smaller parts. For inference, massive high-resolution picture inputs were divided into smaller pieces and fed into the framework. Detection findings from tiny pieces were integrated to create an ultimate high-resolution picture. Furthermore, a multi-morphology augmented data approach was devised and implemented. Initially, faults were extracted using mean filtering with dynamic thresholding against a white or even black backdrop. Augmentation methods, including scaling, mirroring, trimming, rotation, and morphological processing, have been applied to change the geometry of the faults. The procedure resulted in defects that were randomly integrated into fabric pictures in groupings. The findings indicated a mAp of about 75.3%, although only faults among the dataset’s structures were efficiently recognized.

Nasim et al. used a native dataset that is directly obtained by Chenab Textiles, offering genuine and varied photos that accurately depict the circumstances of actual production [1]. The dataset has been utilized for training YOLOv8, a state-of-the-art network that is lighter and computationally quicker. In contrast, the identical dataset is used to train the YOLOv5 along with MobileNetV2-SSD FPN-Lite networks. Having a mAP of about 84.8%, an accuracy of 0.818, along with a recall of about 0.839 over seven distinct defective classes, YOLOv8n demonstrated the best performance. AdaptiveDet is a flaw detection framework for digitally printed fabric that incorporates adaptive attention methods [22]. To better fit the intricate goal form, the K-means++ method was first used to create the first foundation box. Secondly, a flexible CBS module might be used to reorganize the backbone system, reducing interference from non-critical features, thus extracting higher-level characteristics. The ELAN-EVC package was then used to reconstruct the neck structure such that the framework could learn simultaneous globally and locally based feature visualizations to more precisely capture data regarding small flaws. In order to improve the model’s response towards spatial information and achieve exceptional performance during a challenging background defect detection process, the DyHead structure was finally implemented in the header of YOLOv7-Tiny. According to the experiment’s findings, the suggested model outperforms previous detection models by scoring a mAP@.5 of 93% across the DPFD-DET collection. This indicates the system could prove able to satisfy the need for digitally printed fabric’s high-precision fault identification. Since conventional approaches rely on specified defect classifications, they are fundamentally restricted in their ability to detect invisible problems. In order to solve this problem, Ye et al. provide OW-DLN, an open-world fabric defect identification network that unifies generative reconstruction with detached feature learning across known as well as unknown deficiencies to enable efficient detection and continuous learning of undiscovered defects [22]. There are three parts to the design. Semantic learning characteristics are guided through a Mask-Free Inpainting (MFI) section that reconstructs defective pictures towards defect-free image descriptions. A Decoupled Incremental Learning (DIL) section that separates the feature modeling of recognized and unidentified defects, thereby mitigating catastrophic ignorance, while a Dual-View Pseudo-Label Generator (DVPG) module that produces more dependable pseudo-labels, decreasing the omission of unidentified samples, and permitting the framework to more accurately detect features of unforeseen defects. OW-DLN outperforms state-of-the-art (SOTA) OWOD strategies and traditional fabric defect detection techniques in both open-world and incremental scenarios, as demonstrated by extensive experiments utilizing the Tianchi Benchmark alongside a self-collected textile defect dataset. It achieves an undetermined recall of 0.156 while 0.510 mAP@50 within class expansion conditions.

The inference rapidity, localization precision, and detection consistency of existing fabric defect detection techniques are limited. This paper suggests an improved flaw detection approach (Neekolo) determined by You Only Look Once Version 8 (YOLOv8) to solve these issues [23]. Three significant advances are introduced by the suggested framework: To improve the design of the model, an additional detection layer has been incorporated within the feature retrieval network. combined with shadow convolution to reduce feature overlap along with redundant parameters; The design fusion algorithm integrates Omni-dimensional Dynamic Convolution (ODConv) alongside Deformable Large Kernel Attention (DLKA) for enhancing dynamic shape adaptability along with spatial localization by utilizing the morphological, especially distributional features of fabric imperfections; Global-local spatial and channel details are concurrently optimized by a new Neeko attention technique. Both an accessible multi-category defect collection and a uniquely labeled dataset were used for validation purposes. The suggested model considerably speeds up inference while achieving 1.9% while 10.5% gains in 50% (mAP50) across the corresponding datasets contrasted to the benchmark YOLOv8. These findings highlight the model’s capacity for industrial use as well as its resilience to fault variability.

In addition to highlighting a number of present issues [24], investigating ML algorithms within mechanical behavior evaluation of composite substances and applications highlights possible strengths and future directions in the field. When analyzing the mechanical operation of material composites using machine learning, there are several important challenges to solve. Since reliable, high-resolution samples are often necessary for successful model training, material availability as well as quality are significant concerns. However, because comprehensive laboratory testing along with material characterization is required, getting such information sets may be time-consuming and expensive. Additionally, it is difficult for ML methods to accurately represent the complex microstructural variability along with nonlinear component relations seen in material composites. Another problem that can result in algorithms that operate well with standard training data while badly on unforeseen circumstances is overfitting, particularly when working with small datasets. The challenge of describing mathematical models, especially DL methods, makes their wider implementation more difficult, as researchers frequently need to comprehend how forecasts are created. Furthermore, choosing the optimum feature representations and hyperparameters, with methods for particular material systems, continues to be a major technological challenge. Machine learning (ML) is a useful method for determining the mechanical characteristics of composite substances because of its exceptional ability to represent intricate, nonlinear relationships [25]. The fact that these algorithms can be applied to both regulated and unregulated settings shows how versatile they are. ML has also been used to tackle several parametric issues in engineering, especially industrial operations. Several studies have effectively used machine learning techniques to anticipate the mechanical characteristics of different composites. The advancement of reinforcing composite materials has been remarkably accelerated by the application of machine learning modeling techniques.

The literature evaluation revealed two key research gaps. Investigations are divided between printed and ordinary textiles, resulting in separate focal areas. There currently exists no single model that can accurately address both sorts of fabrics. Second, current research focuses on datasets with well-positioned fabric photographs acquired under controlled settings, as shown in Table 1. Real-time manufacturing typically lacks high-quality picture datasets with accurate positioning. Furthermore, enterprises manufacture both simple and patterned textiles. To reliably detect faults in fabric collections during manufacturing, an effective algorithm is needed. Deep learning methods can help overcome these gaps.

**Table 1:** Synthesis matrix with printed textiles literature.

Ref.	Dataset	Models	Research Problem Solved	Accuracy /Performance	Limitations
[23]	Custom built and Tianchi	(Neekolo) based on YOLOv8	Inference speed, detection reliability, and localization accuracy	On custom dataset mAp:96.1 Tianchi mAp:44.8	Not suitable for inter-patch dependencies
[26]	Tianchi and ZJU-Leaper	OW-DLN	Unseen defects by dependence are arranged in predefined defect classes	mAp:0.736 ± 0.009 and mAp:0.708 ± 0.006	Domain-robust or self-adaptive
[22]	DPFD-DET	AdDet	Excellent for the difficult background defect	mAP@.5 of 93%	Lightweight design
[1]	Chenab Textile	Yolo8	Native dataset openly sourced by Chenab Textiles	mAp: 84.8	Real-time testing
[21]	Self-collected dataset with 19 distinct backdrops	Cascade-RCNN	Detection of defects in printed textiles	mAP: 75.3	Effective only for patterns within the dataset
[20]	Tianchi Fabric with Tile Defect Detecting dataset	Siamese FPN	Existing approaches rely heavily on large annotated datasets and fail with unseen samples	mAP: 47.1, Accuracy: 83.3	Requires template images for comparison
[19]	Self-collected(98 fabric drawings)	(DSLRD)	Detecting defects in unevenly printed textiles with complex patterns	TPR: 89.29, FPR: 0.85	Weak robustness across variations
[12]	TILDA dataset	RGBAAM + Image Pyramid	Defect localization via periodic unit identification	Training time reduced by half compared to prior methods	Long processing time for complex designs; may require adaptation in real manufacturing
[9]	Newly proposed dataset	Deep CNN	Classification of placement and print incompatibility defects	Recall: 0.71, Precision: 0.70, Accuracy: 72	Limited to two types of flaws

To reliably detect faults in fabric collections during manufacturing, an effective algorithm is needed. Deep learning methods can help overcome these gaps.

## 3 Proposed methods and materials

This section describes the dataset, incorporating data collection together with preparation processes. The approach of this research and model designs are further discussed.

### 3.1 Data collection and preprocessing

In this work, we trained the proposed model UniDefectNet-Omni with the publicly available Chenab Textile dataset [1,27]. The proposed model UniDefectNet-Omni was then validated with three publicly available datasets, such as Tildav2 [28,29], DPFD-DET [30], and ZJU-Leaper [31]. The Chenab Textile dataset includes printed with plain cloth photos acquired under various production settings. Shortlisting was conducted on the typicality of a certain fault class. Only a few data samples were collected for uncommon defects. Such as between five and six picture samples collected over between four and five months. Consequently, such classes were eliminated. The dataset covers seven categories: baekra, color difficulties, contamination, trimmed, gray stitching, selvet, and stains. Color difficulties include color and discoloration faults. Data preparation involved creating folders for every group and manually adding photographs from different periods. The dataset was then annotated with Roboflow. To accommodate most detection algorithms, including YOLO, researchers manually created square boundaries for all faulty samples. This data set exhibited a class imbalance issue. Certain classifications, such as stain, have significantly larger sample sizes than others. To address this issue, classes with fewer samples received further augmentation. Augmentation methods, such as picture rotation alongside flipping, were widely used. The dataset has been separated into three categories (train, test, alongside valid) while exported using YOLOv12 plus record formats via Roboflow. Initially, model training yielded unsatisfactory mAP readings for several classes. When retraining the model across these classes, image samples from publicly available datasets with Roboflow were used. The total dataset includes around 2800 observations.

### 3.2 Defect categories for the Chenab textile dataset

The study covers seven categories: baekra, color difficulties, contamination, cut, gray stitch, selvet, and stain. Colored difficulties include colored spots and deterioration defects. The dataset comprises instances of plain cloth, frequently printed fabric, and erratically printed fabric. Table 2 shows more about the problems reported by the entire industry.

**Table 2:** Detailed description of defects in the Chenab textile dataset.

Defect Type	Description
Contamination	When a different thread comes making touch with the initial threads of the textile when weaving, it creates a visible imprint on the fabric in the format of a stitch, which is recognized as contamination.
Selvet	When moving, if a single layer of fabric folds slightly, being pushed from the remaining layers holds it in place, creating a folded impression. This defect is called selvet.
Gray Stitch	When a single piece of fabric ends or is ripped by the knitting machine while attaching it to a different one, we employ a stitch called a joint relationship, frequently referred to for its gray stitch.
Cut	This category includes any cut within the cloth resulting from machines or for other reasons. Cuts may appear at the margins of clothing.
Baekra	Whenever the printing process on the machine ceases, it may leave scratches in lines, creating large outages and ruining the design while leaving no pattern. This defect is referred to as baekra.
Color Issues	(1) Inadequate color coverage when printing leads to lighter regions. (2) Printing defects that emerge as spots when color or other debris comes making touch with the fabric when printing. (3) Colors mixing refers to the process of combining two colors.
Stains	Stains might consist of different sorts, including oil, dust, and rust. They might appear as dots or spread out throughout the material.

The total dataset includes around 2800 items. Table 3 displays statistics on the total number of instances for each class within the dataset.

**Table 3:** Annotated data trials for individual class.

Classes	Train	Valid	Test
Baekra	175	48	17
Color issues	86	15	10
Contamination	148	53	14
Cut	272	75	41
Gray stitch	228	63	33
Selvet	218	64	30
Stain	662	208	112

### 3.3 TILDA FabricV2 dataset

The dataset contains 896 images. Every image underwent the pre-processing described below. Fabric is annotated in the YOLOv12 structure. Pixel data is automatically oriented (with EXIF alignment stripped). Resize up to 416 × 416 (Stretching) Grayscale (CCT phosphor). The subsequent augmentation was used to generate three copies of every initial image. The likelihood of a horizontal flip is 50%. The likelihood of a vertical flip is 50%. There is an equal chance of choosing between the subsequent 90-degree alternating: none, clockwise, or counterclockwise [29]. The details of defects are explained in Table 4. The total dataset includes around 896 items. Table 5 displays statistics on the total number of instances for each class within the dataset.

**Table 4:** Detailed description of defects in the Tilda dataset.

Defect Type	Description
Hole	A damaged needle during sewing or the weaving process is a common cause of holes in cloth. This happens when the needle cracks or bends, resulting in an uneven puncture or rip in the material.
Objects	Existence of objects with threads. These errors are difficult to categorize; however, the model distinguishes between them using broad and fine-grained information obtained from convolutional and residual layers, respectively.
Oil spot	Oil spots are among the most confusing faults, as they might appear similar to other problems on the textile surface.
Thread error	Thread defects and imperfect fabric structure occurring during the knitting process.

**Table 5:** Sample detail of TILDA data.

Dataset	Train	Valid	Test
TILDA Fabric v2	776	80	40

### 3.4 Dataset innovation and characteristics

The literature part mentions significant datasets, including TILDA [29,32], TIANCHI [33,34], and AITEX [35], which help identify defects efficiently. However, these photographs are often prepared and of good quality, focused primarily on the fault region without distracting background elements. Our initial dataset includes photos captured immediately from the producing line, lacking any operator placement or modification. The “as-produced” technique includes natural defects and background features from the production process. This dataset provides an authentic training and forecasting context for detecting defects algorithms.

### 3.5 DPFD-DET dataset

Each of the four different forms of defects within colored fabrics, such as Oil, Hole, Cutting, as well as Crack are represented by 720 photographs in the collection, with around 180 images given category. This collection has substantial academic importance, especially in the field regarding computer vision, where it can be an essential tool for creating standard, deep learning, including image processing techniques for tasks like segmentation, object recognition, and classification. The incorporation of it has the potential to greatly accelerate improvements within textile designing and manufacturing techniques. The details of defects are explained in Table 6. The total dataset includes around 720 items. Table 7 displays statistics on the total number of instances for each class within the dataset.

**Table 6:** Detailed defect description of DPFD-DET dataset.

Defect Type	Description
Oil spot	Oil spots are among the most confusing faults, as they might appear to be other problems on the exterior of the textile.
Hole	A damaged needle during the sewing along with the weaving process, is a common cause of holes in cloth. This happens once the needle cracks or bends, resulting in an uneven puncture or rip in the material.
Cutting	Cutting defects may significantly affect the production cycle as a whole, resulting in higher expenses, material waste, and ultimately disgruntled customers. Manufacturers must comprehend the reasons for cutting defects in the clothing sector to apply practical fixes and boost productivity.
Cracks	Starting mark refers to selecting a density that is above usual, whereas fracture refers to a picking density that is lower than normal. Usually loom’s mechanical defects are the primary source of this type of problem.

**Table 7:** Sample detail of DPFD-DET dataset.

Dataset	Train	Valid	Test
DPFD-DET dataset	540	73	107

### 3.6 ZJU-Leaper dataset

ZJU-Leaper, which offers 98,777 fabric photos with detailed annotations from 19 different fabric categories and five groups. Such current fabric datasets, which are made up of individual but non-time-series cloth photographs and were created under idealized fabrication settings, are invaluable resources. In this work, we selected ZJU-Leaper with groups 1, 2, 3, and 4 having 6992 images with twelve defect categories such as ‘broken-end’, ‘coffee-stain’, ‘double-ends’, ‘double-picks’, ‘ink-stain’, ‘knots’, ‘ladder’, ‘missing-picks’, ‘oil-stain’, ‘pin-marks’, ‘slip-knot’, and ‘thread-out’. The details of defects are explained in Table 8. The total dataset includes around 6992 items. Table 9 displays statistics on the total number of instances for each class within the dataset.

**Table 8:** Detailed defect description of DPFD-DET dataset.

Defect Type	Description
Broken-End	Consequence of the surplus yarn weaving within the fabric’s stuffing direction when a warp ending breaks.
Coffee-Stain	The water-soluble, while acidic colored compounds in coffee constitute the primary source of coffee stains regarding fabrics when a warp ending breaks and the extra yarn weaving itself through the fabric’s filled direction.
Double-Ends	An unintentionally doubled thread, particularly a portion of a strand of thread inside the warp.
Double-Picks	The thread, as well as threads in the weaving, that unintentionally double to produce the woven pattern.
Ink-Stain	Stains might consist of different sorts, including oil, ink, dust, and rust. They might appear as dots or spread out throughout the material.
Knots	Knitting represents the technique of interlacing yarns while inter-meshing loops to create knitted cloth.
Ladder	Knitted textiles have this defect. It shows a row with stitches that have been dropped according to the wale standard direction.
Missing-Picks	Omission providing a complete pick over the cloth’s breadth.
Oil-Stain	Stains might consist of different sorts, including oil, ink, dust, and rust. They might appear as dots or spread out throughout the material.
Pin-Marks	These pin spaces are produced while a fabric gets placed across a pin-shaped tenter; the spaces are made far beyond the selvedge, causing the cloth to break or distort.
Slip-Knot	A slipped knot adds an additional tail towards the object.
Thread-Out	Under stress, threads might tear either strip.

**Table 9:** Sample detail of ZJU-Leaper dataset.

Dataset	Train	Valid	Test
DPFD-DET dataset	5394	59	1539

### 3.7 Proposed methodology

**Fig 1:**
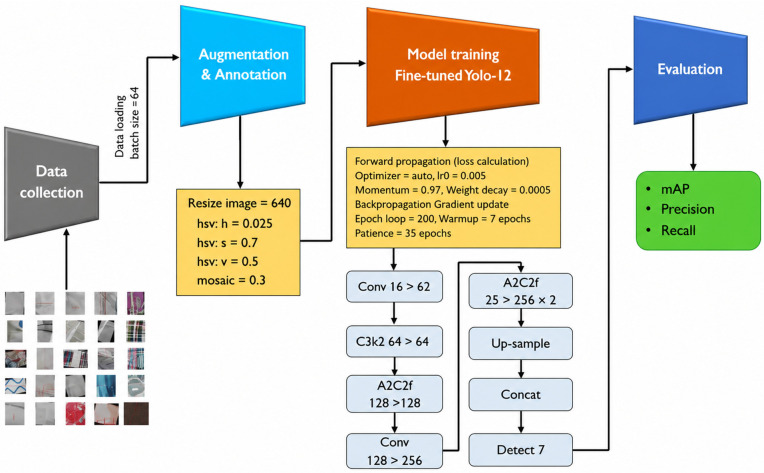
Proposed Yolo12 architecture.

A broad summary of the technique is represented in Fig 1. The dataset gathered by Textile industry with Tilda was enhanced and annotated. The scenario used required speedier designs that were capable of being deployed in low-resource situations. The system has been developed for high-speed fabric manufacturing facilities, where cameras have been carefully placed to scan continually created fabric and spot problems quickly. After reviewing publications, the situation was determined that YOLO represents the most appropriate object identification method for these cases.

YOLO approaches object detection as simply an isolated regression issue. YOLO analyzes the whole image in a single forward run through the algorithm, while other models examine regions that are significant independently, which takes more time. Such an approach minimizes computing costs and delays. The proposed UniDefectNet-Omni maintains the original backbone, detection head, and neck of YOLO-V12, unlike modifying its architecture with additional transformer, attention, or feature fusion components. The proposed solution has the ability of a unified strategy for detection by combining high-resolution training, diverse representations, and adaptive augmentation to enhance the performance of detection across various fabric defect categories, characteristics, and generalization capacity. As a result, YOLOv12 is taught to spot problems. Following model training, projections were produced for test data samples. Adding photos from publicly accessible datasets increased the accuracy of classes with lower mAPs.

#### 3.7.1 Libraries.

The code is implemented in Jupyter Notebook using the Kaggle platform. The specific libraries are imported. The first library is Ultralytics. Which develops innovative (SOTA) YOLO modeling based on numerous years of fundamental research in image processing and Intelligence. They excel with object identification, observing, instance division, image classification, including posture estimation. Squarify provides a Processing module that performs its squarify treemap structuring technique. It splits an area through rectangles, each of which is sized according to a collection of values, with the rectangles designed to be as square as feasible. Matplotlib uses the Pillow package to load picture data. This is simply a 24-bit RGB PNG picture (8 bits per R, G, and B channel). Depending on where receive data, it will also come across RGBA pictures, which enable transparency, and single-channel monochrome (luminosity) images.

#### 3.7.2 Datasets acquisition.

Datasets are split into three sub-directories train, test, and validation. Labels are separated into three sub-directories. Before training the machine vision model, it requires labeled information. The accuracy of the labels, called annotations, determines the performance of the model. Datasets with train, test, and validation sets can be defined in Equation 1–3.


$$\begin{array}{c} {\text{Data}}_{\text{train}}=\sum_{i=1}^{N_{\text{train}}}(t_{i},tl_{i}) \end{array}$$ (1)

$$\begin{array}{c} {\text{Data}}_{\text{valid}}=\sum_{i=1}^{N_{\text{valid}}}(v_{i},vl_{i}) \end{array}$$ (2)

$$\begin{array}{c} {\text{Data}}_{\text{test}}=\sum_{i=1}^{N_{\text{test}}}(te_{i},tel_{i}) \end{array}$$ (3)

where $t_{i},v_{i},te_{i}$ are images in training, validation, and test sets, $tl_{i},vl_{i},tel_{i}$ are the corresponding labels, and $N_{\text{train}},N_{\text{valid}},N_{\text{test}}$ are the number of samples in each set.

#### 3.7.3 Trying Yolo-12 on pre-trained images.

**Fig 2:**
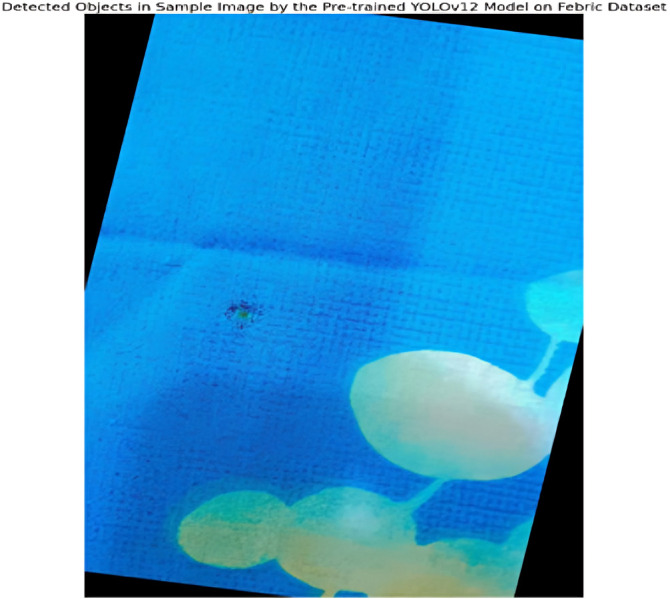
Yolo-12 on pre-trained images using the Chenab textile dataset.

**Fig 3:**
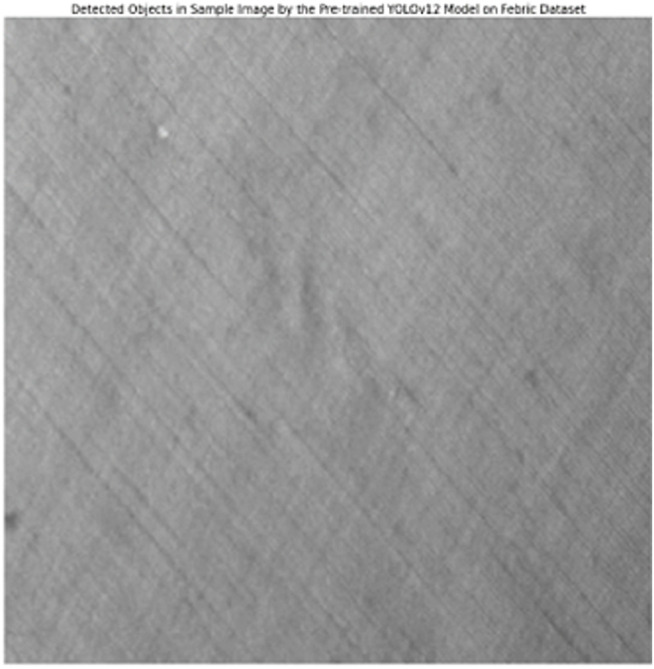
Yolo-12 on pre-trained images using Tildav2 dataset.

**Fig 4:**
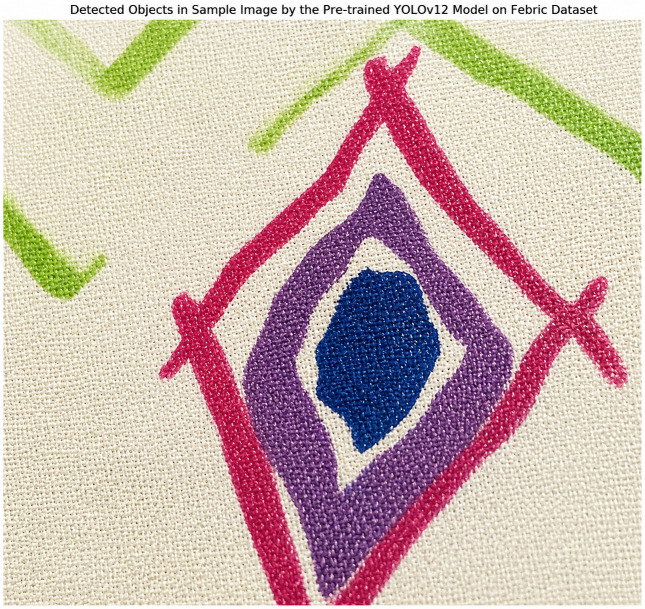
Yolo-12 on pre-trained images using the DPFD dataset.

**Fig 5:**
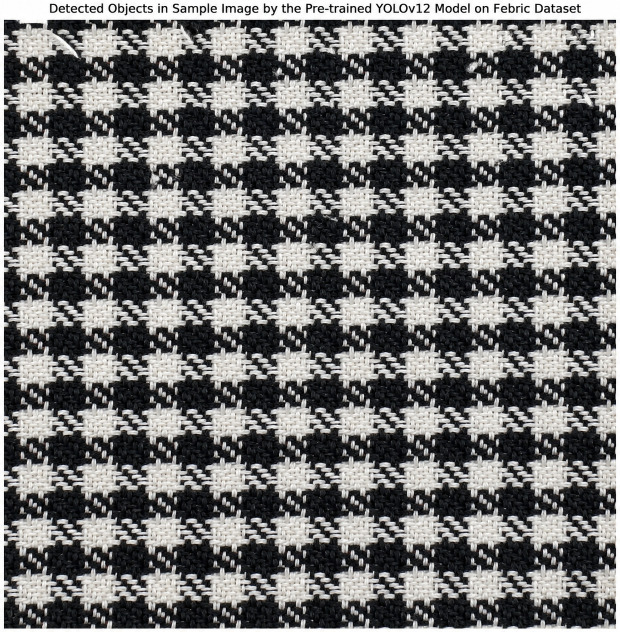
Yolo-12 on pre-trained images using the ZJU-Leaper dataset.

In the next step, we tried defect detection on pre-trained test images of both datasets. The images in Figs 2–5 show that no defect is detected in the image from all datasets.

#### 3.7.4 Hyperparameters adjustment.

proposed model was quick, accurate, and simple to use, while they are regularly revised to improve performance and versatility. Various hyperparameter configurations were utilized to train the YOLOv12 networks. The standard hyperparameter choices for YOLOv12, including image dimensions 640, and periods 200, alongside batch size 64, yielded optimal results. The beginning and end learning rates were 0.005 with 0.01, respectively, with a momentum equal to 0.97 with weight decay equal to 0.0005. To help sustain the training sessions, a 7-epoch warming-up phase is also included. The following YOLO settings were used:hsv_h = 0.025, hsv_s = 0.7, hsv_v = 0.5, mosaic = 0.3. Dropout is set to 0.025 as Deactivates a subset of the neural networks (units) within that layer during each training session. This indicates that the chosen neurons’ outputs have been configured to zero, therefore they have no impact on the subsequent pass during the back-propagation process for that particular sample. Patience is set to 35 for epochs wait for no obvious progress before discontinuing training. Optimizer is set to auto for auto adjustment of optimization through the training process. Similar was the case with yolov12. It was cloned using ultrasonics. A “data.yaml” file exported in YOLOv12 format from roboflow was provided to the model for training. YOLOv12 was trained for 200 epochs and an image size of 640 with default parameters to produce approximately similar results; hence, YOLOv12 was chosen as the final model. All key equations representing parameters are as follows Equations 4–11.


$$\begin{array}{c} \text{Learning Rate}(ep)=lr_{i}\left(\frac{1+\cos\left(\pi \frac{ep}{F_{ep}}\right)}{2}\right)\times (1-l_{f})+l_{f} \end{array}$$ (4)

Here learning Rate(ep) at epoch ep. lri is the initial learning rate = 0.005. lrf is final learning rate = 0.01 and Fep are total epochs = 200 for Textile industry dataset and 1 for Tildav2.


$$\begin{array}{c} \text{Momentum update with optimizer:}\;mo=\mu \times mo+\text{lrc}\times \nabla G_{L}(\theta_{l}) \end{array}$$ (5)

Where μ is momentum = 0.97, lrc is the current learning rate,$\nabla G_{L}(\theta_{l})$ is loss in Gradient and θ is the models weight. The adjusted equation is as under


$$\begin{array}{c} \mu_{0}^{+}=0.97\mu_{0}^{-}+lrc\times \nabla G_{L}(\theta_{l}) \end{array}$$ (6)

$$\begin{array}{c} L_{\text{total}}=L_{\text{org}}+0.0005\times \theta^{2} \end{array}$$ (7)

Here *L*2_total_ is total regularization and ω is weight decay = 0.0005.


$$\begin{array}{c} \text{Dropout Regularization (DR)}(x)=f(x)\cdot m,\;m\sim \text{Bernoulli}(1-0.025) \end{array}$$ (8)

Where do is dropout = 0.025


$$\begin{array}{c} \text{Warm-up Learning Rate (WULR)}(ep)=lr_{i}\cdot \frac{ep}{E_{wulr}} \end{array}$$ (9)

Where $lr_{i}$ denotes the initial learning rate at epoch *ep*. The adjusted equation is as follows:


$$\begin{array}{c} {\text{Learning Rate}}_{\text{warm}}(ep)={\text{LR}}_{i}\cdot \frac{ep}{7},\;0\leq ep\leq 7. \end{array}$$ (10)

$$\begin{array}{cccc} \text{Augmentation(HSV and Mosaic)}H^{\prime } & =H+\sigma_{H};S^{\prime } & =S\times (1+\sigma_{S});V^{\prime } & =V\times (1+\sigma_{V}) \end{array}$$ (11)

$$\begin{array}{c} \sigma_{H}\sim 𝒰(-0.025,\;0.025),\;\sigma_{S}\sim 𝒰(-0.7,\;0.7),\;\sigma_{V}\sim 𝒰(-0.5,\;0.5). \end{array}$$

Mosaic combined four images in one for a broader context. Saturation transformation


$$\begin{array}{c} ST^{\prime }=ST\left(1+\Delta ST\right),\;\Delta ST\in [-0.7,\;0.7] \end{array}$$ (12)

Value transformation


$$\begin{array}{c} VT^{\prime }=VT\left(1+\Delta VT\right),\;\Delta VT\in [-0.5,\;0.5] \end{array}$$ (13)

Mosaic augmentation


$$\begin{array}{c} {\text{Img}}_{\text{mosaic}}=M\left({\text{Img}}_{1},{\text{Img}}_{2},{\text{Img}}_{3},{\text{Img}}_{4}\right) \end{array}$$ (14)

where *P*(Img_mosaic_) = 0.3.

**Training** Jupyter Notebook through Kaggle, having a T4 GPU and 16GB GPU memory, was used for training, while inference was performed together with the Ultralytics architecture. The data set output by roboflow using the yolov12 format includes a “data.yaml” item with information on any number of categories and training, testing, and validation folders. Ultralytics was used to configure the YOLOv12 packaging, and the algorithm received a “data.yaml” file as its training data. The image size and the total number of intervals were specified as 640 by 200, respectively. The average training time was approximately 8 hours.

## 4 Results and discussion

This section presents the experimental results and provides a comprehensive discussion of the findings. The proposed approach is evaluated based on precision and recall, and the outcomes are compared with state-of-the-art methods to highlight its strengths. Mean average precision, mostly written as mAP, is used mainly as an evaluation metric in this study. However, along with mAP, recall(R) and precision(P) have also been displayed for UniDefectNet-Omni. The details of results generated by UniDefectNet-Omni are as overall mAp50 for the Chenab textile dataset is 85.1%, for the Tilda v2 dataset is 86.7%, for the DPFD dataset is 93.6%, and for the ZJU-Leaper dataset is 93%. Similarly, mAp95 for the Chenab textile dataset is 57.5%, for the Tilda v2 dataset is 59.5%, for the DPFD dataset is 60.1%, and for the ZJU-Leaper dataset is 51.5%. The individual class accuracy for the Chenab textile dataset shows remarkable results for contamination class with mAP of 98.7%, stain with mAP 93.4%, cut with mAP 81.6%, Baekra with mAP 85%, Selvet with mAP 84.7%, color issues with mAP 71.7%, and gray stitch with mAP 80.9%. Precision and recall values for all classes are 84.5% and 81.7%. The values can be viewed in Table 10.

**Table 10:** Class-wise performance of YOLOv12 on the Textile industry dataset.

Class	mAP@0.5	mAP@0.5:0.95
All	0.851	0.575
Baekra	0.850	0.700
Color issues	0.717	0.457
Contamination	0.987	0.581
Cut	0.816	0.533
Gray stitch	0.809	0.574
Selvedge	0.847	0.556
Stain	0.934	0.625

The individual class precision for the Tilda v2 dataset shows results for the oil spot class with a mAp of 98.9%, objects with mAp of 91.8%, hole with mAp of 87.4%, and thread error with mAp 68.5%. Precision and recall values for all classes are 83.7% and 83.6%. The values can be viewed in the Table 11.

**Table 11:** Class-wise performance of YOLOv12 on the Tildav2 dataset.

Class	mAP@0.5	mAP@0.5–0.95
All	0.867	0.595
Hole	0.874	0.604
Objects	0.918	0.629
Oil spot	0.989	0.749
Thread error	0.685	0.397

The individual class accuracy for the DPDF dataset shows results for the oil spot class with mAP of 98.9%, hole with mAP of 97%, cutting with mAP of 85.4%, and cracks with mAP 93%. Precision and recall values for all classes are 92.2% and 87.8%. The values can be viewed in Table 12.

**Table 12:** Results of Yolo12 on the DPFD dataset.

Class	mAP@0.5	mAP@0.5–0.95
All	0.936	0.601
Oil spot	0.989	0.667
Hole	0.97	0.743
Cutting	0.854	0.438
Cracks	0.93	0.559

The individual class accuracy for the ZJU-Leaper dataset shows results for Broken-End, Coffee-Stain, Ink-Stain, Ladder, Oil-Stain, Pin-Marks, Slip-Knot, and Thread-Out classes with mAP of 99.5%, Double-Ends with mAP of 85.3%, Double-Picks with mAP of 91.6%, Knots with mAP of 76.1%, and Missing-Picks with mAP 67.2%. Precision and recall values for all classes are 78.1% and 90.3%. The values can be viewed in Table 13.

**Table 13:** Results of Yolo12 on the ZJU-Leaper dataset.

Class	mAP@0.5	mAP@0.5–0.95
All	0.93	0.515
Broken-End	0.995	0.471
Coffee-Stain	0.995	0.883
Double-Ends	0.853	0.408
Double-Picks	0.916	0.503
Ink-Stain	0.995	0.255
Knots	0.761	0.268
Ladder	0.995	0.504
Missing-Picks	0.672	0.279
Oil-Stain	0.995	0.735
Pin-Marks	0.995	0.753
Slip-Knot	0.995	0.554
Thread-Out	0.995	0.569

### 4.1 The learning curve for box loss for the Textile industry dataset

**Fig 6:**
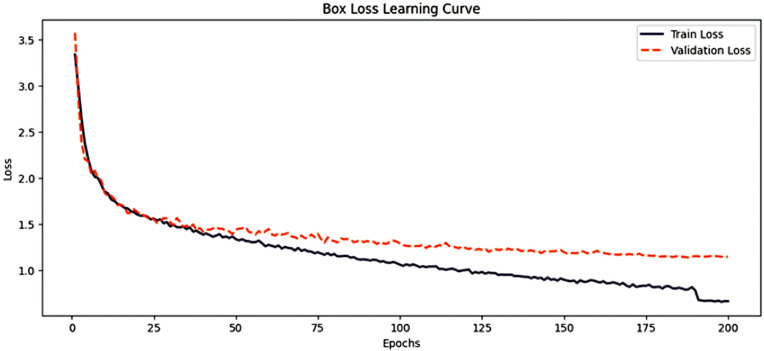
Box loss for Chenab Textiles dataset.

A learning curve provides a representation showing model performance during learning across experiences or time. Figure 6 below shows that training and validation loss gradually decreased for the Textile industry dataset across epochs or during model training.

### 4.2 The learning curve for Classification loss for the Textile industry dataset

**Fig 7:**
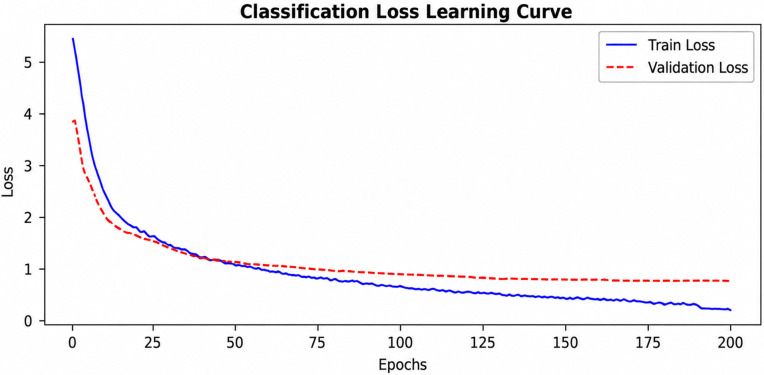
Classification loss for Chenab Textiles dataset.

The classification loss graph represents model learning for data classification over training. Figure 7 below represents classification loss for the Textile industry dataset. Classification loss is gradually decreasing for training and validation. This shows showing the model is accurately trained and validated.

### 4.3 The learning curve for distribution focal loss for the Textile industry dataset

**Fig 8:**
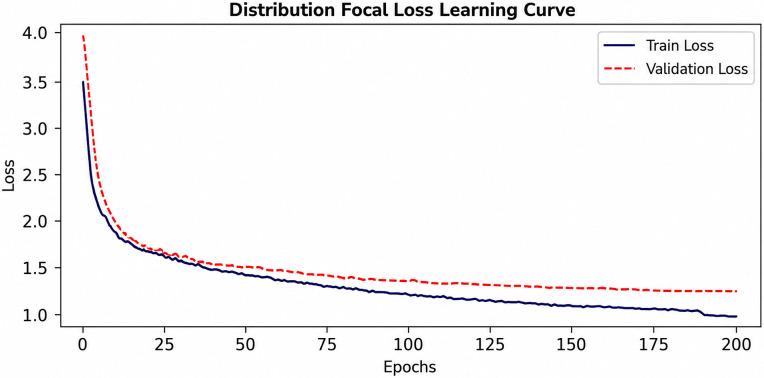
Distribution of focal loss for Chenab Textiles dataset.

Using the Distribution Focal Loss algorithm solves class imbalance during classification with semantic segmentation by elaborating on the Focal Loss functional concepts. Focal Loss increases losses for details with a big discrepancy between anticipated and actual products, thereby forcing neural networks to focus on more difficult-to-classify cases. This is especially effective in cases of class imbalance. Figure 8 shows showing the distribution focal loss is decreasing. It means the Textile industry dataset is properly trained and data loss is also decreasing.

### 4.4 Confusion matrix for Textile industry dataset

**Fig 9:**
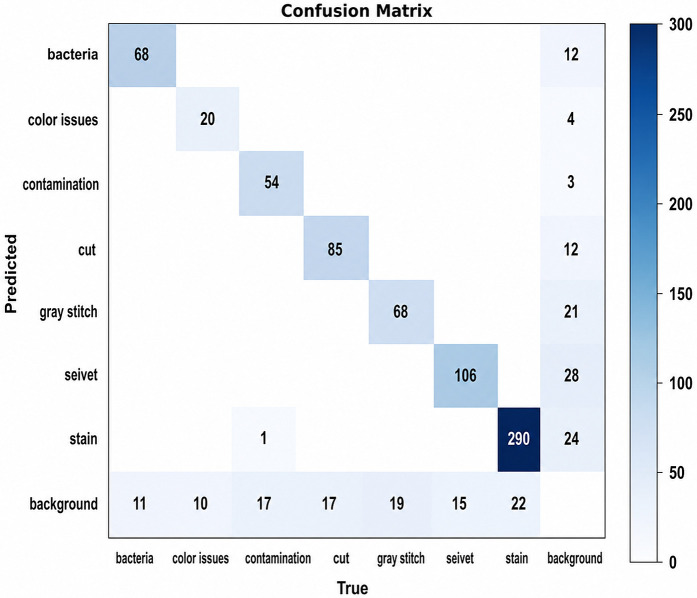
Confusion matrix for Chenab Textiles dataset.

A confusion matrix includes a table designed to describe the effectiveness of a classification method. A confusion matrix depicts and analyzes the performance of a technique for classification. The confusion matrix in Fig 9 represents 68 samples of baekra class that are true and predicted true and 12 samples are misclassified. The 20 samples of color issues class true classified and 4 are misclassified. The 54 samples of contamination class are truly classified and 3 are misclassified. The 85 samples of cut class are truly classified and 12 are misclassified. The 68 samples of gray stich class are true and truly predicted with 21 are misclassified. The 106 samples of selvet class are truly classified and 28 are misclassified. The 290 samples of stain class are truly predicted and 24 are misclassified. Defects having low contrast, irregular boundaries, and a small size failed to activate discriminative structures; the model interpreted them as background. Similarly, texture variations, wrinkles, illumination changes, or noise resemble a defect pattern learned as a defect-free region. The model can be overfit with local features. The class stain has large samples, which limits feature generalization.

### 4.5 Validation inferences for the Textile industry dataset

**Fig 10:**
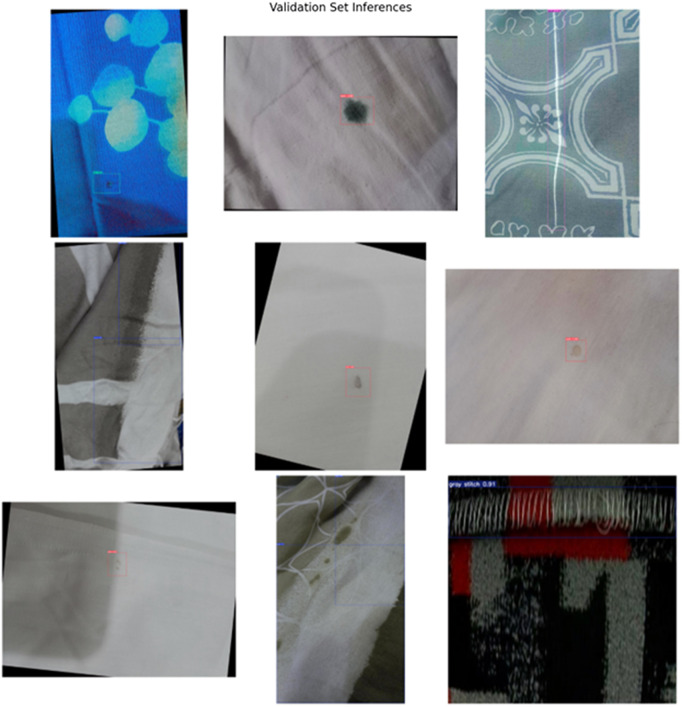
Validation inferences for Chenab Textiles dataset.

The following Fig 10 shows defect detection on the Textile industry fabric dataset’s validation data. Red boxes indicate predicted defects and blue for reality-based truth annotations. Several images are narrowly localized defects truly detected with labeled using appropriate boxes. The model tackles both structural and surface defects. Both large and small defects are detected by the model. It indicates appropriate performance for multi-scale datasets.

### 4.6 Detected object through fine-tuned Yolo-12 for Textile industry dataset

**Fig 11:**
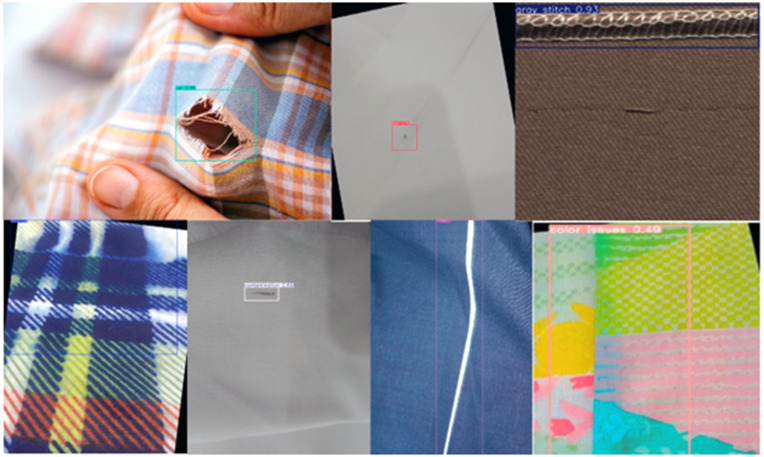
Detected object through fine-tuned Yolo-12 for Chenab Textiles dataset.

The detection result in Fig 11 shows that the confidence score of the cut defect is 88%. It means our Fine-tuned Yolo-12 is considered a State-of-the-art detection model.

### 4.7 The learning curve for box loss for the Tilda dataset

**Fig 12:**
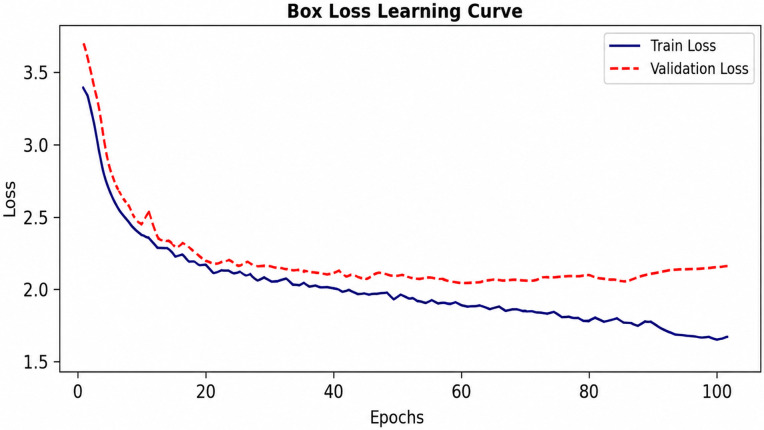
Box loss for the Tilda dataset.

A learning curve provides a representation showing model performance during learning across experiences or time. Fig 12 below shows that training and validation loss gradually decrease for the Tildav2 dataset across epochs or during model training.

### 4.8 The learning curve for the Classification loss for the Tilda dataset

**Fig 13:**
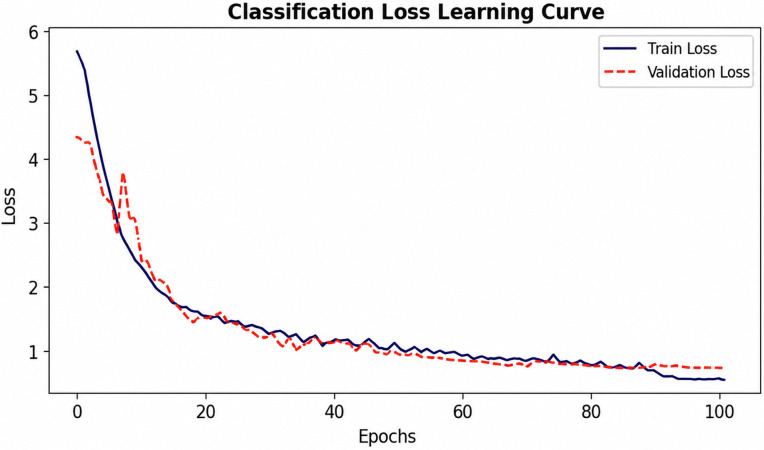
Validation inferences for the Tilda dataset.

The classification loss graph represents model learning for data classification over training. Fig 13 below represents classification loss for the Tildav2 dataset. Classification loss is gradually decreasing for training and validation. This shows showing the model is accurately trained and validated.

### 4.9 The learning curve for distribution focal loss for the Tilda dataset

**Fig 14:**
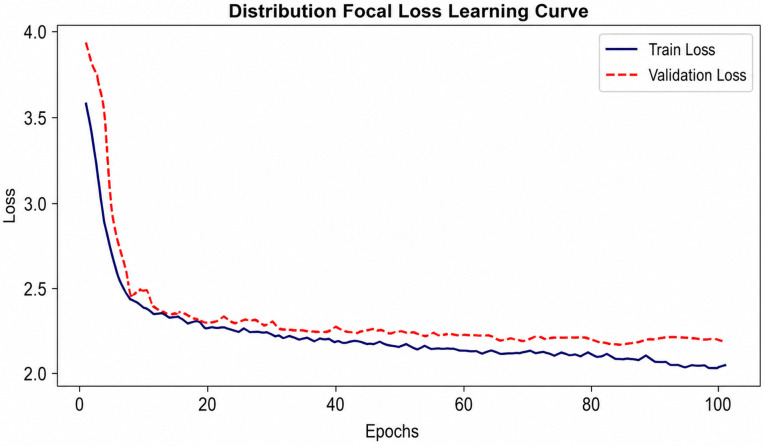
Distribution of focal loss for the Tilda dataset.

Using the Distribution Focal Loss algorithm solves class imbalance during classification with semantic segmentation by elaborating on the Focal Loss functional concepts. Focal Loss increases losses for details with a big discrepancy between anticipated and actual products, thereby forcing neural networks to focus on more difficult-to-classify cases. This is especially effective in cases of class imbalance. Fig 14 shows that the focal loss in the distribution is decreasing. It means the Tildav2 dataset is properly trained and data loss is also decreasing.

### 4.10 Confusion matrix for Tilda dataset

**Fig 15:**
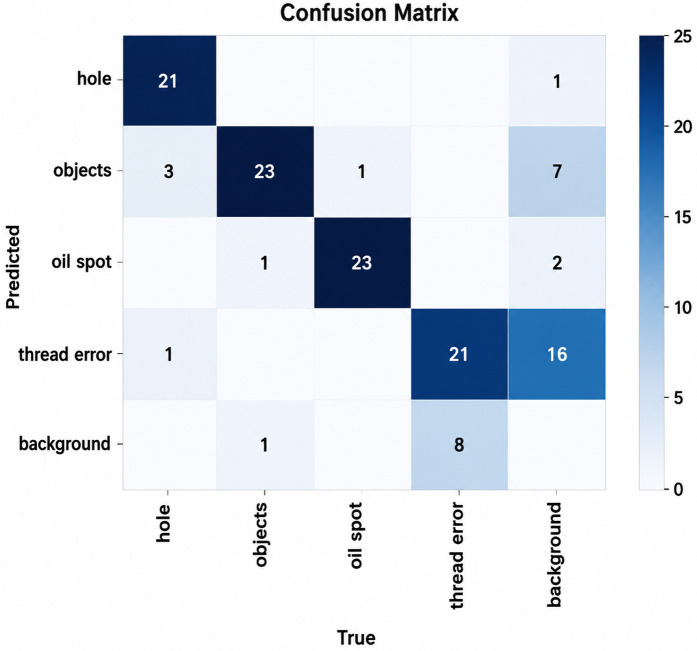
Confusion matrix for the Tilda dataset.

A confusion matrix includes a table designed to describe the effectiveness of a classification method. A confusion matrix depicts and analyzes the performance of a technique for classification. The confusion matrix in Fig 15 represents 21 samples of hole class that are true and predicted true and 1 samples are misclassified. Of the 23 samples of objects class is true classified and 11 are misclassified. The 23 samples of oil spot class are truly classified and 3 are misclassified. The 21 samples of thread error class are truly classified and 17 are misclassified. The thread errors are very similar to fabric structures. The extracted features can be overlapped with regular patterns. Similarly, holes and objects may have edge structures with occlusion effects.

### 4.11 Validation inferences for the Tilda dataset

**Fig 16:**
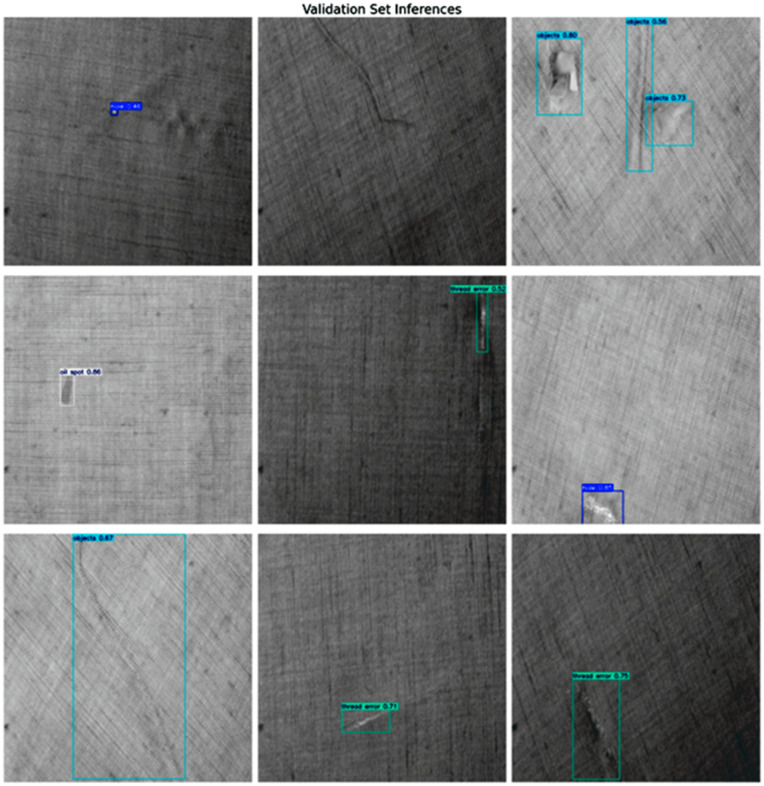
Validation inferences for the Tilda dataset.

The following Fig 16 shows defect detection on the Tildav2 fabric dataset’s validation data. Boxes indicate predicted defective and reality-based truth annotations. Several images are narrowly localized defects truly detected with labeled using appropriate boxes. The model tackles both structural and surface defects. Both large and small defects are detected by the model. It indicates appropriate performance for multi-scale datasets.

### 4.12 Detected object through fine-tuned Yolo-12 for Tilda dataset

**Fig 17:**
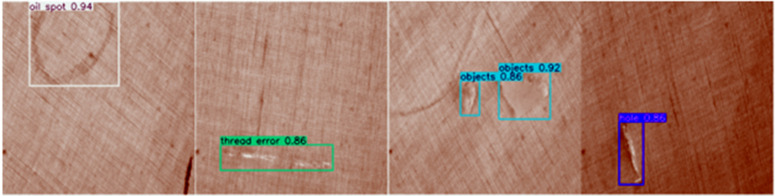
Detected object through fine-tuned Yolo-12 for the Tilda dataset.

The detection result in Fig 17 shows the confidence score of oil spot defect is 84%. It means our Fine-tuned Yolo-12 is considered a State-of-the-art detection model.

### 4.13 The learning curve for box loss for the DPFD dataset

**Fig 18:**
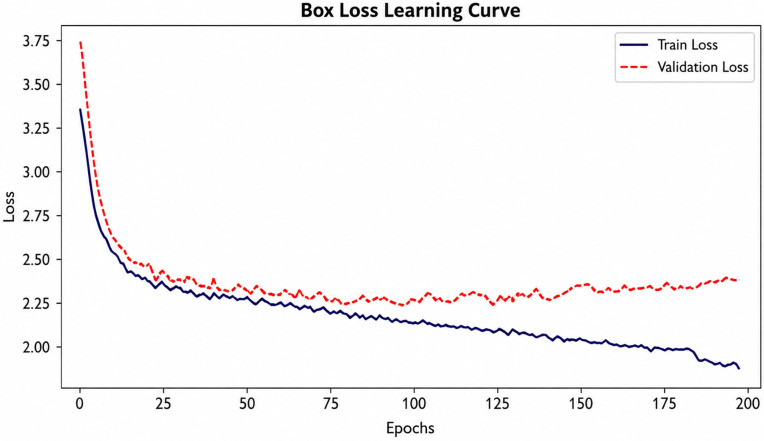
Box loss for DPFD dataset.

A learning curve provides a representation showing model performance during learning across experiences or time. Fig 18 below shows that training and validation loss gradually decrease for the DPFD dataset across epochs or during model training.

### 4.14 The learning curve for the classification loss for the DPFD dataset

**Fig 19:**
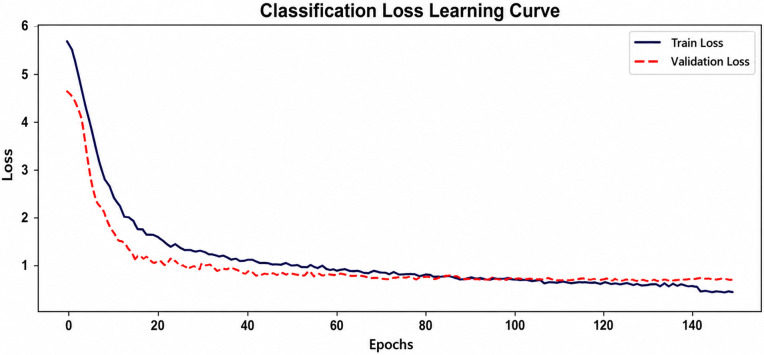
Classification loss for the DPFD dataset.

The classification loss graph represents model learning for data classification over training. Fig 19 below represents the classification loss for the DPFD dataset. Classification loss is gradually decreasing for training and validation. This shows showing the model is accurately trained and validated.

### 4.15 The learning curve for distribution focal loss for the DPFD dataset

**Fig 20:**
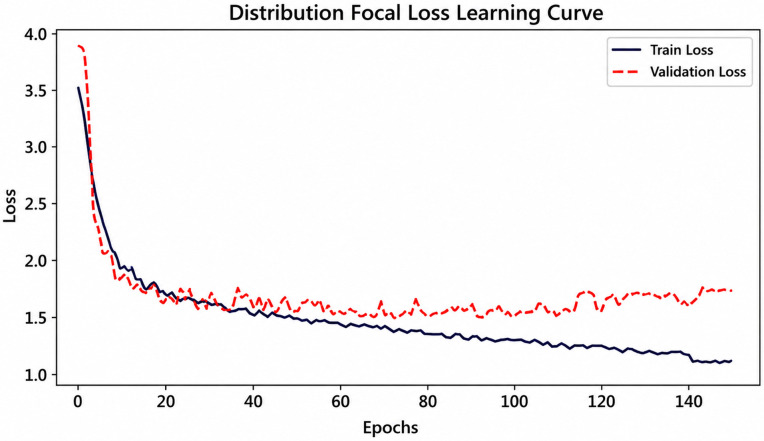
Distribution of focal loss for the DPFD dataset.

Using the Distribution Focal Loss algorithm solves class imbalance during classification with semantic segmentation by elaborating on the Focal Loss functional concepts. Focal Loss increases losses for details with a big discrepancy between anticipated and actual products, thereby forcing neural networks to focus on more difficult-to-classify cases. This is especially effective in cases of class imbalance. Fig 20 shows that the distribution of focal loss is decreasing. It means the DPFD dataset is properly trained, and data loss is also decreasing.

### 4.16 Confusion matrix for DPFD dataset

**Fig 21:**
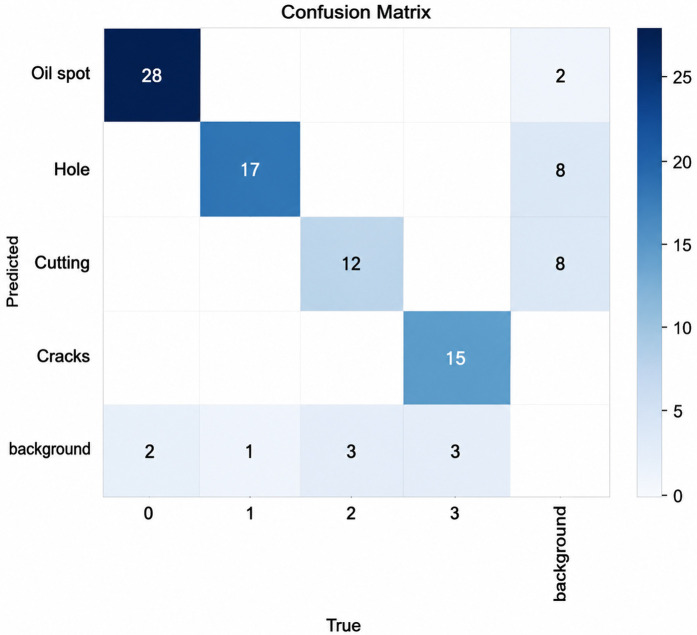
Confusion matrix for the DPFD dataset.

A confusion matrix includes a table designed to describe the effectiveness of a classification method. A confusion matrix depicts and analyzes the performance of a technique for classification. The confusion matrix in Fig 21 represents 28 samples of the oil-spot class that are true and predicted true, and 0 samples are misclassified. The 17 samples of hole class are truly classified, and 2 are misclassified. The 12 samples of the cutting class are truly classified, and 8 are misclassified. The 15 samples of the cracks class are truly classified, and 0 are misclassified. The wide samples are concentrated along the a diagonal, showing strong class discrimination. The small number of Hole = 1, Oil spot = 2, Crack = 3, and Cutting = 3 instances were categorized as background. It suggested that some regions of defects were not appropriately distinctive for reliable detection.

### 4.17 Validation inferences for the DPFD dataset

**Fig 22:**
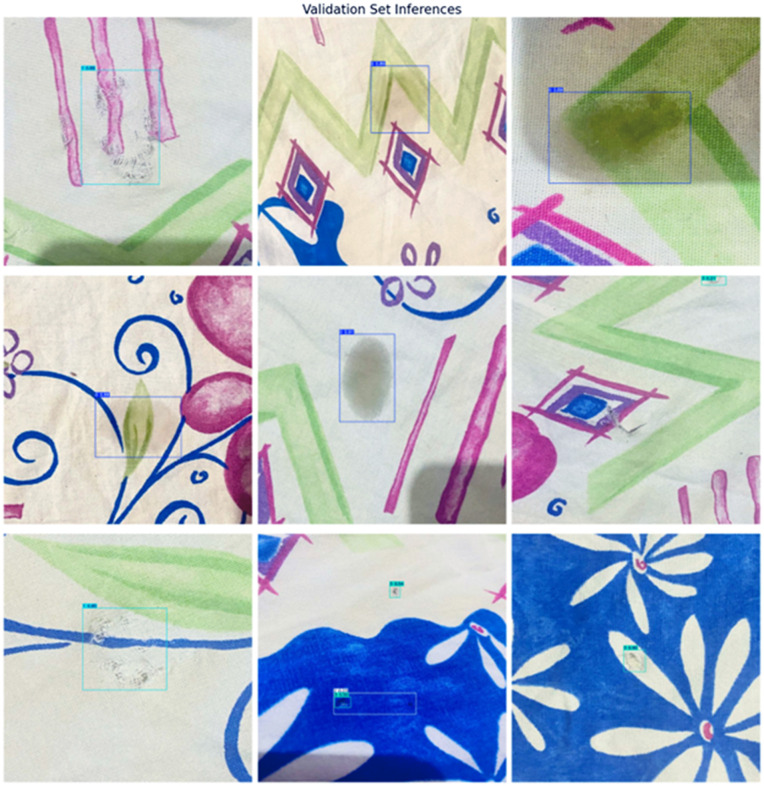
Validation inferences for the DPFD dataset.

The following Fig 22 shows defect detection on the DPFD dataset’s validation data. Boxes indicate predicted defective and reality-based truth annotations. Several images are narrowly localized defects truly detected with labeled using appropriate boxes. The model tackles both structural and surface defects. Both large and small defects are detected by the model. It indicates appropriate performance for multi-scale datasets.

### 4.18 Detected object through fine-tuned Yolo-12 for DPFD dataset

**Fig 23:**
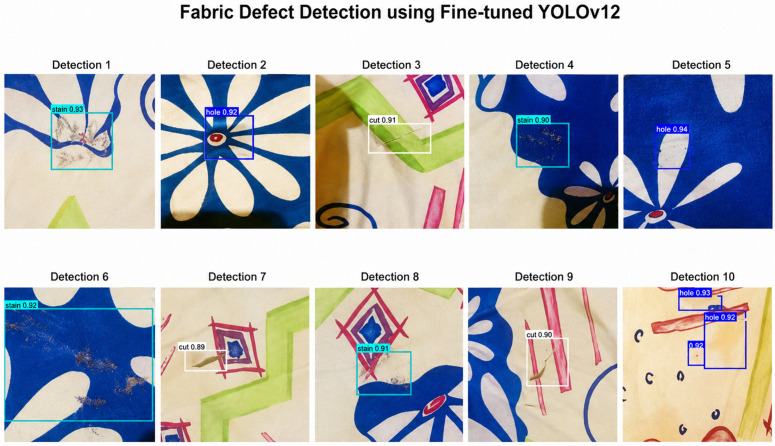
Detected object through fine-tuned Yolo-12 for DPFD dataset.

The detection result in Fig 23 shows the confidence score of the oil spot defect is 91%. It means our Fine-tuned Yolov-12 is considered a State-of-the-art detection model.

### 4.19 The learning curve for box loss for the ZJU-Leaper dataset

**Fig 24:**
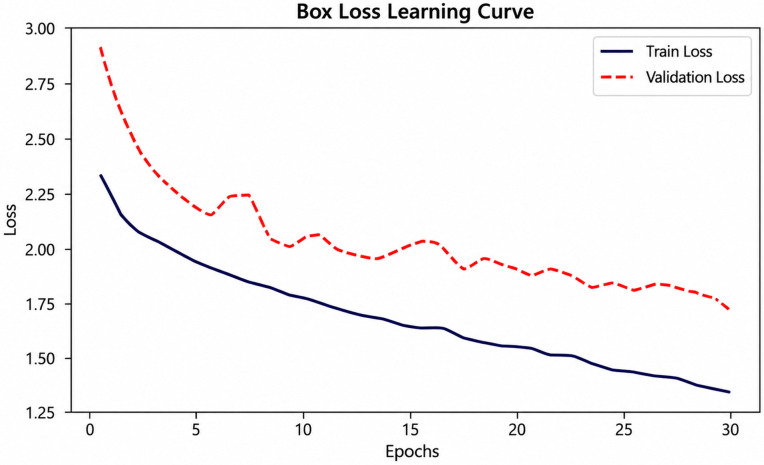
Box loss for ZJU-Leaper dataset.

A learning curve provides a representation showing model performance during learning across experiences or time. Fig 24 below shows that training and validation loss gradually decrease for the ZJU-Leaper dataset across epochs or during model training.

### 4.20 The learning curve for the classification loss for the ZJU-Leaper dataset

**Fig 25:**
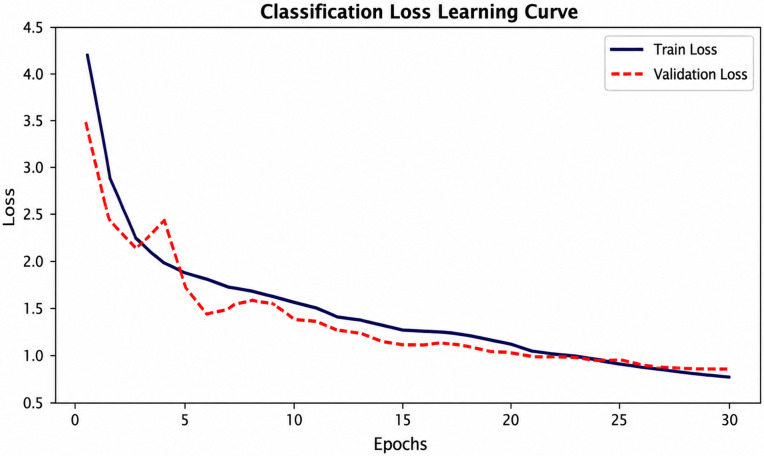
Classification loss for the ZJU-Leaper dataset.

The classification loss graph represents model learning for data classification over training. Fig 25 below represents the classification loss for the ZJU-Leaper dataset. Classification loss is gradually decreasing for training and validation. This shows showing the model is accurately trained and validated.

### 4.21 The learning curve for distribution focal loss for the ZJU-Leaper dataset

**Fig 26:**
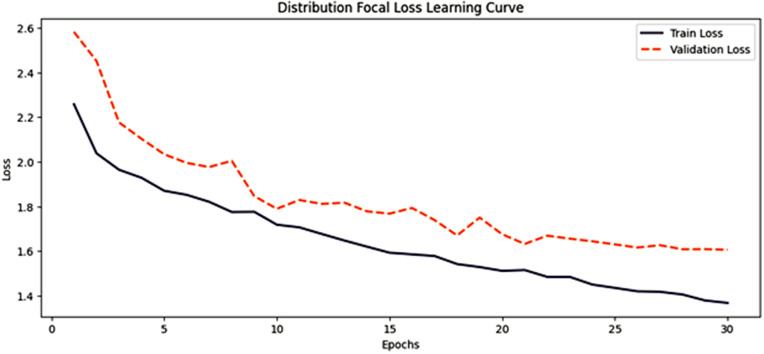
Distribution of focal loss for the ZJU-Leaper dataset.

Using the Distribution Focal Loss algorithm solves class imbalance during classification with semantic segmentation by elaborating on the Focal Loss functional concepts. Focal Loss increases losses for details with a big discrepancy between anticipated and actual products, thereby forcing neural networks to focus on more difficult-to-classify cases. This is especially effective in cases of class imbalance. Fig 26 shows that the distribution of focal loss is decreasing. It means the ZJU-Leaper dataset is properly trained, and data loss is also decreasing.

### 4.22 Confusion matrix for ZJU-Leaper dataset

**Fig 27:**
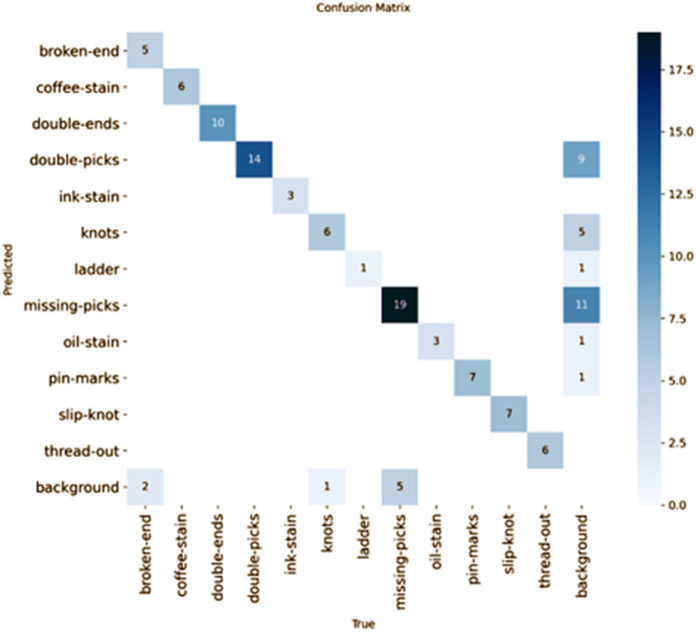
Confusion matrix for the ZJU-Leaper dataset.

A confusion matrix includes a table designed to describe the effectiveness of a classification method. A confusion matrix depicts and analyzes the performance of a technique for classification. The confusion matrix in Fig 27 represents 5 samples of broken-end class that are true and predicted true, and 0 samples are misclassified. The 6 samples of coffee-stain class are truly classified, and 0 are misclassified. The 10 samples of double-ends class are truly classified, and 0 are misclassified. The 14 samples of the double-picks class are truly classified, and 9 are misclassified. The 3 samples of ink-stain class are truly classified, and 0 are misclassified. The 6 samples of konts class are truly classified, and 5 are misclassified. The 1 sample of ladder class is truly classified, and 1 is misclassified. The 19 samples of the missing-picks class are truly classified, and 11 are misclassified. The 3 samples of oil-stain class are truly classified, and 1 is misclassified. The 7 samples of pin-marks class are truly classified, and 1 is misclassified. The 7 samples of slip-kont class are truly classified, and 0 are misclassified. The 6 samples of thread-out class are truly classified, and 0 are misclassified. The dominant error source is samples with background, specifically Knots, Broken-End, and Missing picks. This indicates that the model is suitable for defect detection but weak for visual contrast, small spatial level, irregular boundaries, or textures. The feature representations learning is highly discriminative. The remaining errors were associated with defect sensitivity and defect localization instead of class ambiguity.

### 4.23 Validation inferences for the ZJU-Leaper dataset

**Fig 28:**
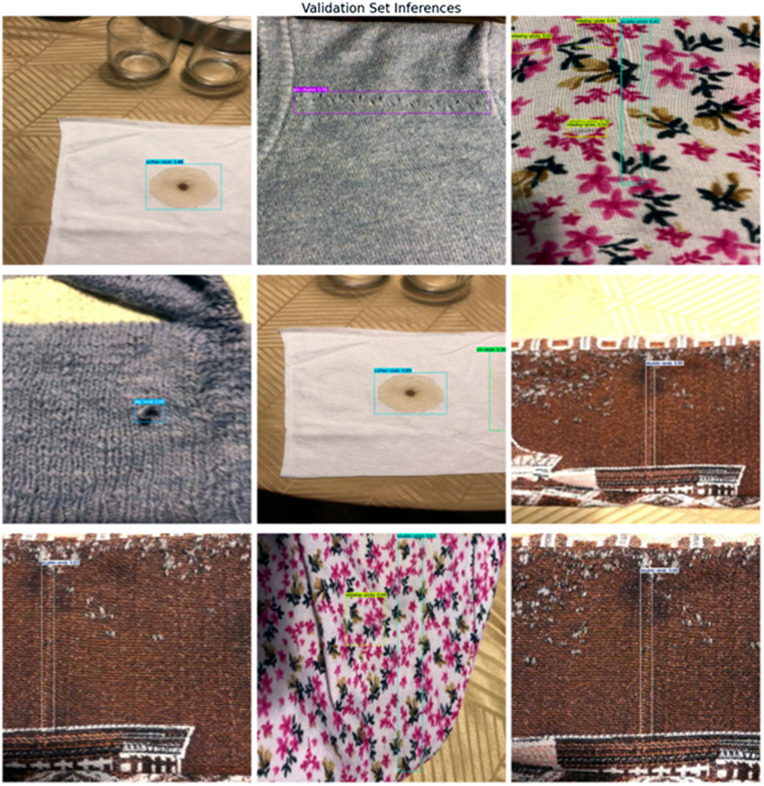
Validation inferences for the ZJU-Leaper dataset.

The following Fig 28 shows defect detection on the ZJU-Leaper dataset’s validation data. Boxes indicate predicted defective and reality-based truth annotations. Several images have narrowly localized defects that are truly detected and labeled using appropriate boxes. The model tackles both structural and surface defects. Both large and small defects are detected by the model. It indicates appropriate performance for multi-scale datasets.

### 4.24 Detected object through fine-tuned Yolo-12 for ZJU-Leaper dataset

**Fig 29:**
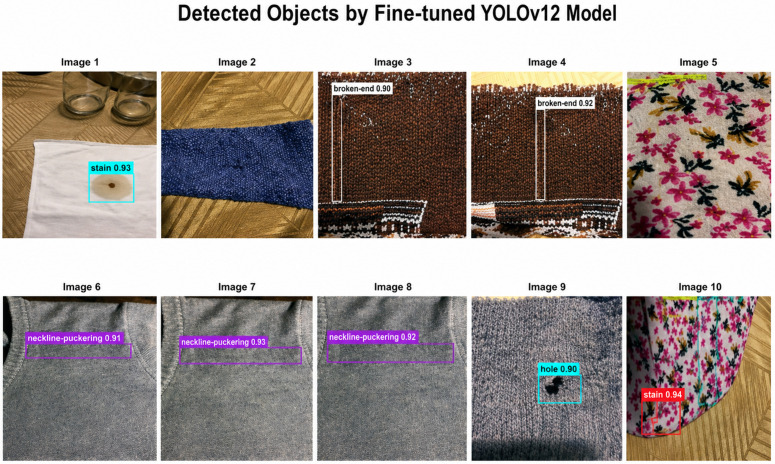
Detected object through fine-tuned Yolo-12 for ZJU-Leaper dataset.

The detection result in Fig 29 shows the confidence score of the coffee-stain defect is 88%. It means our Fine-tuned Yolo-12 is considered a State-of-the-art detection model.

### 4.25 Discussion

**Fig 30:**
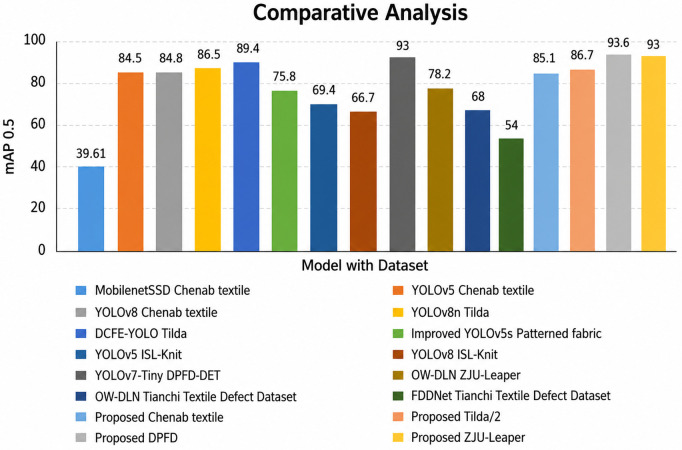
Comparative analysis with state of arts methods.

The proposed UniDefectNet-Omni proves strong defect detection ability with diverse defects, such as colored, grayscale, plain, and printed fabrics. The conventional YOLO defect detectors only optimized specific fabrics or limited fabric defect categories. Whereas UniDefectNet-Omni is a unified solution for defect-invariant representations for diverse fabrics. The proposed model is successful for contamination, structural, and texture-related defects with a single model with stabilizing computational efficiency. While transformer-based frameworks such as OW-DLN and AdaptiveDet introduced supplementary architectural complexity. UniDefectNet-Omni attains competitive performance with operative feature learning and generalization of defect representation, making it more appropriate for across-the-board industrial inspection. While comparing with the recent study [26], the proposed UniDefectNet-Omni has improved consistency for stain and structural defect detection. Unlike [23], that highlight global accessible fields, UniDefectNet-Omni has balanced augmentation, learning ability for high resolution, and effective small-scale defect localization, such as thread-out, pin marks, and cracks. The work of [26] focused on open-world flexibility proposed UniDefectNet-Omni, which is stable without complex decoupled learning approaches. Furthermore, comparing with [22] which only targeted complicated printed fabrics, the proposed work maintains modest performance with both simple and high-textured backgrounds, proving strong generalization ability. While comparing mAp levels from several past investigations in Fig 30.

Table 14 presents a comprehensive comparison of the proposed model with state-of-the-art approaches across multiple textile defect datasets using mAP@0.5 as the evaluation metric. UniDefectNet-Omni achieved higher results, achieving an aggregate mAP of about 85.1% across the Chenab textile sample, 86.7% for the Tildav2 sample, 93.6%, for the DPFD sample, and for ZJU-Leaper with groups 1, 2, 3, and 4 having 93%. UniDefectNet-Omni outperformed other algorithms in contamination, baekra, selvet, and stain detection in Chenab textile dataset. Favorable outcomes were achieved with high percentages regarding cut, color difficulties, and gray stitching. In Tildav2 objects defects achieved 91.8% and oil spot is 98.9% detected. These are also favorable results. In DPFD dataset oil-spot achieved 98.9% detection and hole detection is 97%, that is also high performance indication. In ZJU-leaper dataset, most of defect classes accurately detected and model outperformed current studies. The results are 99.5% for Broken-End, Coffee-Stain, Ink-Stain, Ladder, Oil-Stain, Pin-Marks, Slip-Knot and Thread-Out. In certain courses, UniDefectNet-Omni was surpassed. This strategy improves accuracy. This conclusion shows the complexities of techniques since performance advances in certain areas might not correlate to overall benefits.

**Table 14:** Performance comparison of defect detection models on textile datasets.

Model	mAP@0.5	mAP@0.5:0.95	Precision	Recall	Fitness	Dataset	Ref.
MobileNetSSD	77.09	39.61	52.5	88.1	–	Chenab textile	[1]
YOLOv5	84.5	52.5	82.7	84.5	–	Chenab textile	[1]
YOLOv8	84.8	57.5	81.8	83.9	–	Chenab textile	[1]
YOLOv8n	86.5	46.9	83.8	81.5	–	Tilda	[2]
DCFE-YOLO	89.4	49.0	87.3	83.7	–	Tilda	[2]
Improved YOLOv5s	75.80	–	–	–	–	Patterned fabric	[3]
YOLOv5	69.4	35.1	77.7	65.2	–	ISL-Knit	[4]
YOLOv8	66.7	35.2	73.3	63.2	–	ISL-Knit	[4]
YOLOv7-Tiny	93.0	68.2	87.2	85.2	–	DPFD-DET	[22]
OW-DLN	73.6 ± 0.0009	–	–	–	–	ZJU-Leaper	[26]
OW-DLN	68.0 ± 0.009	–	–	–	–	Tianchi Dataset	[26]
FDDNet	54.0	27.9	56.1	54.3	–	Tianchi Dataset	[36]
**YOLO-V12 (UniDefectNet-Omni)**	**85.1**	**57.5**	**84.5**	**81.7**	**60.3**	Chenab textile	–
**YOLO-V12 (UniDefectNet-Omni)**	**86.7**	**59.5**	**83.7**	**83.6**	**62.2**	Tilda v2	–
**YOLO-V12 (UniDefectNet-Omni)**	**93.6**	**60.1**	**92.2**	**87.8**	**60.1**	DPFD	–
**YOLO-V12 (UniDefectNet-Omni)**	**93.0**	**51.5**	**78.1**	**90.3**	**51.2**	ZJU-Leaper	–

The detection of faults was accurate, with well-defined boundary lines and high confidence ratings. The degree of trust for the threshold class within the test specimen is quite high, with values of 0.88 for the Chenab textile collection, 0.84 within Tildav2, 0.91 in the DPFD dataset, and 0.88 in the ZJU-Leaper dataset. The newest object identification model, UniDefectNet-Omni, may identify faults in both plain and patterned textiles, including regular and uneven designs. The results indicate that serious problems may be accurately discovered. The mAp frequency “thread error” within the Tildav2 and Missing-Picks in ZJU-Leaper datasets seems rather low. To improve, consider including additional data samples within that course to ensure effective pattern learning. The current analysis focuses on a limited number of typical problems within Pakistan’s textile business and relies only on producer data. Adding more variants to the collection can help increase accuracy.

#### 4.25.1 Ablation study for adjusted hyper-parameter.

The adjusted training parameters for UniDefectNet-Omni were justified through an ablation analysis on the Chenab Textile Dataset. The varied hyperparameters were applied, and other settings remained unchanged. The mAP@50 is a major performance evaluation metric. Table 15 shows ablation results.In this Table presents the impact of different training configurations on model performance in terms of mAP@50%. Several hyperparameters were systematically varied, including input image size, learning rate, Mosaic augmentation ratio, and warmup epochs.

**Table 15:** Effect of different training configurations on model performance.

Config	Image Size	LR	Mosaic	Warmup Epochs	mAP@50%
A	640	0.001	0.3	3	82.8
B	640	0.003	0.3	5	83.5
C	640	0.005	0.3	7	85.1
D	640	0.007	0.3	7	83.7
E	512	0.005	0.3	7	82.6
F	768	0.005	0.3	7	84.8
G	640	0.005	0.4	7	83.9

The outcomes indicated the learning rate of 0.005 achieved the top detection accuracy having stable convergence. High learning rates showed oscillatory training performance, whereas lower rates presented optimization. The resolution 640 × 640 showed effective stability for computational cost and feature preservation. The generalization improved through restrained mosaic augmentation by revealing the model for diverse patterns of defects. The warm-up trained with 7 epochs is useful for stabilizing the gradient during the early stages of optimization. On the basis of these hit-and-trial methods, adjusted hyperparameters were configured and adopted for experiments.

## 5 Conclusions and future work

This work proposed UniDefectNet-Omni, a unified defect detection solution for plain and printed fabrics. The proposed model is capable of accurately identifying diverse defects such as structural, texture-oriented, and contamination. The proposed UniDefectNet-Omni achieves high mAP across diverse datasets and explores its robustness. The results clarify that optimal training with a scalable architecture can efficiently explore small size defect, background complexity, and texture variations. Our method includes training and evaluating UniDefectNet-Omni, a substance detection model, using the Chenab Textile, Tildav2, DPFD, and ZJU-Leaper datasets. YOLO-V12 / UniDefectNet-Omni was chosen for its computing speed and resource efficiency for defect detection. UniDefectNet-Omni achieved a mAp of around 85.1% for the Chenab textile collection, which included samples for 7 defect categories (stains, cut, contamination, bakery, gray thread, color problems, and selvet). During the second testing for 4 defect types (hole, oil spot, thread fault, and objects), UniDefectNet-Omni achieved an mAp equivalent to 86.7% for the Tildav2 dataset. In the third DPFD dataset, the mAP score 93.6%, with four defect classes, such as Oil, Hole, Cutting, and Crack. ZJU-Leaper with groups 1, 2, 3, and 4 having mAP score 93% over twelve separate fabric defect categories such as ‘broken-end’, ‘coffee-stain’, ‘double-ends’, ‘double-picks’, ‘ink-stain’, ‘knots’, ‘ladder’, ‘missing-picks’, ‘oil-stain’, ‘pin-marks’, ‘slip-knot’, and ‘thread-out’. Despite high performance, the proposed UniDefectNet-Omni has certain limitations. The detection may face issues for low-contrast images, such as jeans fabrics. The model is not incorporating transformer mechanisms, which may enhance feature representation. Similarly, exclusion of rare defects to reduce class imbalance and training stability may affect the model’s generalization for unseen defects.

We plan to test this framework in real time within high-speed textile manufacturing scenarios. To incorporate the model’s training into existing production procedures, sophisticated cameras using high-speed internet connections will be mounted above the moving sheets. Bright illumination will ensure excellent image quality. This machine will operate on Ubuntu or Windows, using a deep learning platform installed to configure the environment. The trained UniDefectNet-Omni modeling weights shall be loaded while set up for real-time interpretation. A database would be required to store detection findings. Visualization libraries may be used to provide a user experience that displays real-time outcomes of detection, including fault analysis, as long as the system runs smoothly. Furthermore, an explainable defect detection model will be trained and validated for deeper defect analysis. The structure can be incorporated with camera-based inspection in manufacturing. Such automation may reduce manual reliance. Early defect detection will reduce material waste, rework cost, and improve production. Similarly, early detection can contribute to the rejection rate, customer satisfaction, and lower quality costs.
