# Supplementary material for: Fabric defect detection using fine-tuned Yolo-12
Source: PLoS One. 2026 Jul 22;21(7):e0353550. doi: 10.1371/journal.pone.0353550 (PMC13390829; doi:10.1371/journal.pone.0353550)
Supplement: Attachment — Submitted filename: Response to Reviewers.pdf [file pone.0353550.s003.pdf]

Response to Reviewers

Reviewer #1:

Comments-1:The novelty of UniDefectNet-Omni should be more clearly articulated, particularly in comparison with existing YOLO-based and transformer-based defect detection models.

Reply-1:. Thanks for the reviewer valuable suggestion. In response, we have further clarified novelty of UniDefectNet-Omni's distinctive defect representation architecture enables a single YOLOv12-based model to accurately detect a broad spectrum of textile faults across different fabric qualities, including plain, printed, multicolored, and grayscale textiles. UniDefectNet-Omni demonstrates dependable and consistent performance across a range of heterogeneous textile datasets while maintaining the efficiency required for industrial deployment, in contrast to transformer-based approaches that rely on computationally demanding attention mechanisms and existing YOLO-based models that are usually tested on a single fabric domain. Furthermore, compared with transformer-based defect detection models that rely on computationally intensive attention mechanisms and require higher computational resources, UniDefectNet-Omni maintains a favorable trade-off between detection accuracy, robustness, and computational efficiency, making it suitable for real-time industrial textile inspection The novelty has been included in the comparison on page # 44.

Comments-2: The title is appropriate, but the manuscript inconsistently refers to the model as UniDefectNet-Omni, YOLOv12, and Yolo-12, which should be standardized throughout.

Reply-2:. We thank the reviewer for highlighting this inconsistency. The proposed framework has been consistently referred to as **UniDefectNet-Omni** throughout the revised manuscript, while **YOLOv12** has been used exclusively to denote the underlying backbone architecture. All inconsistent references, including variations such as "YOLO-12" and other naming discrepancies, have been carefully corrected to ensure uniform terminology and improve the overall clarity and consistency of the manuscript.

Comments-3: The abstract should explicitly describe the architectural modifications introduced beyond the baseline YOLOv12 model.

Reply-3:. We thank the reviewer for this important observation. The abstract has been revised to more clearly articulate the nature of the proposed improvements with respect to the baseline YOLOv12 framework. The abstract has been revised to clarify that the proposed approach, which builds on the conventional YOLOv12 architecture, enhances fault representation through dataset variety, high-resolution learning, and improved training procedures rather than architectural change.

Comments-4: The literature review is extensive, but it is overly verbose and contains numerous grammatical errors that obscure the main research gap.

Reply-4:. We sincerely thank the reviewer for this valuable suggestion. The literature review section has been thoroughly revised to improve clarity, conciseness, and grammatical accuracy. Redundant and overly verbose content has been removed, and the discussion has been streamlined to better highlight the key research gap and positioning of the proposed work within the existing literature. The revised section now presents a more focused and coherent narrative aligned with the objectives of the study.

Comments-5: Author should add more about machine learning in literature review section. You may refer "Review of machine learning approaches for predicting mechanical behavior of composite materials", Investigation of Tribological Behaviour of B4C–Graphene Glass Fiber Composites Using Taguchi, "Multi-Criteria Decision Making, and Machine Learning Approaches", "Predictive modeling of thermoplastic nanocomposites using machine learning algorithms".

Reply-5:. The relevant studies, "Review of machine learning approaches for predicting mechanical behavior of composite materials" and Predictive modeling of thermoplastic nanocomposites using machine learning algorithms have been included.

Comments-6: Table 1 is useful, but several performance metrics are reported inconsistent and should be verified against the cited references.

Reply-6: We thank the reviewer for this careful observation. Table 1 has been thoroughly reviewed and cross-checked against the original cited references to ensure accuracy and consistency of all reported performance metrics. The table has been improved and corrected.

Comments-7: The methodology does not clearly explain what architectural changes distinguish UniDefectNet-Omni from the standard YOLOv12 implementation.

Reply-7: We appreciate the reviewer's comment. The suggested system, which is based on the conventional YOLOv12 detector, aims to increase the robustness of defect identification by using adaptive augmentation, high-resolution feature learning, improved training techniques, and heterogeneous textile fault representation. The updated approach makes it clear that UniDefectNet-Omni's value is not in architectural redesign but rather in its unified learning framework and defect generalization capacity. The paper has been revised to provide a clear distinction between the suggested training and assessment framework and the baseline YOLOv12 architecture.

Comments-8: The removal of rare defect classes may limit the generalization capability of the proposed model and should be discussed as a limitation.

Reply-8: We appreciate the reviewer's insightful comment. The article now includes a restriction paragraph that acknowledges that the model's capacity to generalize to occasional or undetectable faults in actual textile production may be diminished by the absence of exceedingly rare defect types.

Comments-9: The statement that additional public images were added after observing poor class performance introduces potential data leakage and should be carefully clarified.

Reply-9: We thank the reviewer for this important concern. We would like to clarify that no additional images were introduced into the dataset during the experiments in a manner that could lead to data leakage or bias. The statement in the manuscript refers solely to the description of the original dataset construction process as reported in prior work. It was included for completeness and context, not as part of any modification to the dataset used in our experiments. All experiments in this study were conducted using the publicly available dataset as-is, without any alteration, augmentation of external images, or class-balancing through additional data collection. We have revised the manuscript to clarify this point more explicitly to avoid any misunderstanding regarding dataset integrity and experimental validity. This statement is refered to original dataset that how dataset built. We just describe dataset creation as reference. We did not add anything to dataset.

Comments-10: Hyperparameter selection appears empirical, and no ablation study is provided to justify the chosen settings.

Reply-10: We value the reviewer's insightful comment. The selected hyperparameters were first determined using a variety of exploratory tests and recommendations from the YOLOv12 training framework. We acknowledge that the text did a poor job of explaining the logic behind these choices. To alleviate this concern, an ablation study has been incorporated to evaluate the impact of significant hyperparameters, including learning rate, batch size, picture quality, data augmentation intensity, and warm-up epochs. The results demonstrate that the selected design provides the greatest feasible balance between convergence stability, detection accuracy, and processing efficiency. The ablation analysis and associated discussion are now included in the revised text.

Comments-11: Several equations in the methodology section are standard definitions and do not add substantial value; instead, more implementation details should be provided.

Reply-11: We value the reviewer's wise suggestion. We have revised the method section by removing unnecessary textbook-level equations to highlight implementation-specific designs, including the true training scheduling configuration, optimizer actions, and parameter settings used in this YOLOv12-based approach. Additional details with learning rate schedules,

momentum configuration, and execution-level learning technique have been incorporated to improve repeatability and simplicity.

Comments-12: Equation 8 contains a notation inconsistency where the same variable is used on both sides of the equation.

Reply-12: We sincerely appreciate the reviewer's perceptive evaluation. We agree that the prior version of Equation 8, now Equation 9, created a notation inconsistency that might lead to misconceptions by using the symbol "wulr" as both the function output and part of the variable name. To overcome this challenge, we have revised the equation using a commonly used and comprehensible mathematical language. The warm-up learning rate is now described as a function of the epoch, and the warm-up length is represented by a separate parameter.

Comments-13: The manuscript should specify the computational resources used for training, including GPU model, memory, and total training time.

Reply-13: We are grateful for the reviewer's important recommendation. We have also given a detailed description of the computational architecture used for model training in order to improve experimental reproducibility and transparency. The Kaggle platform's default GPU setup was used for each experiment. Specifically, the model was trained on a Kaggle GPU T4 accelerator with about 16 GB of GPU RAM. For the training process, Kaggle laptops were utilized rather than an external high-performance computer cluster. The total training time for the proposed model, including the stages of data loading, augmentation, and assessment, was about 8 hours.

Comments-14: Results are presented only for the proposed model without direct benchmarking against baseline models such as YOLOv8, YOLOv11, or Faster R-CNN under identical conditions.

Reply-14: The proposed model is trained on the Chenab textile dataset and validated with three datasets, such as TildaV2, DPFD-DET, and ZJU-Leaper. The model is comprehensively validated.

Comments-15: Furthermore, a detailed comparative analysis is performed in Table 14.The claim that the model is "state-of-the-art" is not justified solely by showing one prediction example with high confidence.

Reply-15: The proposed solution has the ability of a unified strategy for detection by combining high-resolution training, diverse representations, and adaptive augmentation to enhance the performance of detection across various fabric defect categories, characteristics, and generalization capacity. According to a comprehensive review of literature, and to the best of our knowledge, there is no such model that addresses such defect detection through a single model.

Comments-16: Confidence scores of individual detections do not constitute evidence of superior model performance.

Reply-16: We appreciate the reviewer's insightful comments. We concur that better model performance cannot be inferred only from the confidence scores of individual detection cases. The qualitative detection numbers provided a visual representation of the model's capacity to locate and identify textile flaws under typical circumstances. We use quantitative indicators, including accuracy, recall, F1-score, mAP@0.5, mAP@0.5:0.95, confusion matrix analysis, and loss convergence behavior to assess performance objectively. We have explicitly stated in the updated publication that the confidence levels displayed in qualitative examples are just meant for visualization and should not be taken as proof of the overall superiority of the model. To prevent such misunderstandings, corresponding explanation materials have been provided.

Comments-17: The comparison with prior studies should use consistent datasets and evaluation metrics to ensure fairness.

Reply-17: We sincerely thank the reviewer for this important suggestion. The comparative study has been carefully revised to improve fairness, consistency, and methodological rigor. We have ensured that all reported comparisons are aligned in terms of datasets and evaluation metrics, or clearly annotated where differences exist due to variations in original study settings. In addition, the revised manuscript explicitly standardizes the evaluation criteria used for benchmarking, enabling a more reliable and transparent comparison with prior work. These

improvements strengthen the validity and interpretability of the reported results. The comparative study has been updated and improved.

Comments-18: The ZJU-Leaper dataset table is incorrectly labeled as "DPFD-DET dataset" and should be corrected.

Reply-18: We thank the reviewer for carefully reviewing the manuscript.In revised manuscript, we have coreected and updated as per your suggestion.

Comments-19: The manuscript reports exceptionally high mAP values for several classes (0.995), which may indicate overfitting and should be discussed.

Reply-19: We value the reviewer's informative feedback. We acknowledge that concerns regarding potential overfitting may arise from abnormally high mAP values for some fault classes. However, because these classes correspond to flaws with well-recognizable visual characteristics and sufficient training data, they are rather simple to identify. To decrease overfitting during training, regularization techniques such as weight decay, dropout, data augmentation, and early stopping were employed. We do agree, though, that exceptionally high class-wise mAP values may partially reflect the dataset

Comments-20: The confusion matrix analysis is descriptive but does not provide deeper insight into the causes of misclassification.

Reply-20: We appreciate the reviewer's insightful comments. We recognize that excessively high mAP values for some fault classes may raise questions about possible overfitting. However, these classes are rather easy to detect as they correlate to defects with easily identifiable visual traits and enough training data. Regularization strategies such as weight decay, dropout, data augmentation, and early halting were used to reduce overfitting during training. However, we acknowledge that extraordinarily high class-wise mAP values might be a partial reflection of the dataset.

Comments-21: No inference speed (FPS) or latency results are provided, despite claims of suitability for real-time industrial deployment.

Reply-21: We appreciate the reviewer bringing this significant problem to our attention. We concur that when assessing whether a defect detection approach is appropriate for real-time industrial deployment, inference speed and latency are crucial factors. The original publication did not provide thorough runtime benchmarking because the present study's primary focus is on detection accuracy and resilience across varied textile fault datasets.

Comments-22: Cross-dataset generalization is claimed, but no transfer learning or domain adaptation experiments are presented.

Reply-22: We appreciate the reviewer's insightful comment. We concur that transfer learning, domain adaptation, or cross-domain assessment experiments in which a model trained on one dataset is assessed on another dataset without retraining are usually necessary for thorough validation of cross-dataset generalization. The phrase "cross-dataset" was used in the current study to denote that the suggested framework was assessed on many textile defect datasets with various imaging circumstances and defect characteristics.

Comments-23: The practical significance of the proposed approach for textile manufacturers should be discussed in terms of deployment feasibility and economic impact.

Reply-23: In order to emphasize the usefulness of the suggested framework for textile manufacturing settings, we have extended the conclusion in the updated manuscript. In particular, we address how automated defect identification may eliminate material loss related to faulty fabric rolls, enhance product quality consistency, enable early detection of production problems, and lessen the need for manual inspection. We value the reviewer's wise suggestion.

Comments-24: Figures and tables should be carefully proofread for labeling inconsistencies and formatting issues.

Reply-24: We sincerely thank the reviewer for this valuable suggestion. All figures and tables have been carefully re-examined and thoroughly proofread in the revised manuscript. Labeling inconsistencies, formatting issues, and minor typographical errors have been corrected to ensure clarity, uniformity, and professional presentation throughout the document. These revisions improve the overall readability and quality of the manuscript.

Comments-25: The manuscript contains extensive grammatical and typographical errors and requires substantial English language editing.

Reply-25: The complete manuscript has been reviewed, and grammatical errors have been removed.

Comments-26: Overall, the study is promising and industrially relevant, but major revisions are required to clarify the methodological novelty, strengthen comparative evaluation, and improve the presentation quality before publication.

Reply-26: We truly appreciate the reviewer's favorable evaluation of the study and helpful suggestions. We have made significant revisions to the paper in response. Initially, we explained the methodological contribution of the suggested UniDefectNet-Omni framework and made a clear distinction between its contributions and changes made to the underlying YOLOv12 detector's architecture. Second, we went into more detail about the experimental findings, constraints, capacity for generalization, confusion matrix analysis, and industrial relevance. Third, by updating graphics, tables, methodological explanations, mathematical formulations, and result interpretations, we enhanced the overall quality of the presentation.

Reviewer #2:

Comments-1: The manuscript addresses a highly relevant industrial problem by proposing a unified defect detection framework (UniDefectNet-Omni) capable of identifying defects in both plain and printed fabrics, which is a notable contribution given that most previous studies focus on only one fabric category.

Reply-1: We truly appreciate the reviewer's favorable evaluation of our work. The limits of current textile defect detection techniques, which are frequently created and assessed for a particular fabric type, were one of the main driving forces for the creation of UniDefectNet-Omni. The suggested method seeks to increase resilience across various textile inspection scenarios and improve its practical application in industrial settings by integrating both plain and printed fabric defect datasets under a single detection framework. We are grateful that the reviewer acknowledged this contribution, and in the updated text, we have highlighted its industrial value and rationale even more. To facilitate defect identification in both plain and printed fabric categories, a uniform framework for textile defect detection called UniDefectNet-Omni is suggested. The suggested framework is intended to function across diverse textile inspection scenarios, which enhances its practical applicability for industrial quality-control applications in contrast to many current systems that concentrate on a particular fabric type.

Comments-2: The evaluation on multiple datasets (Chenab Textile, TILDA v2, DPFD-DET, and ZJU-Leaper) demonstrates the model's generalization capability and provides comprehensive validation across diverse textile defect types.

Reply-2: The resilience and usefulness of the suggested UniDefectNet-Omni framework across varied textile inspection situations are demonstrated by the assessment carried out on many textile defect datasets, including Chenab Textile, TILDA v2, DPFD-DET, and ZJU-Leaper. The model's efficacy in managing a broad range of textile defect patterns is demonstrated by the experimental findings, which offer thorough validation over various defect characteristics, fabric textures, and imaging settings. Dedicated cross-dataset transfer studies are still an option for future research to further explore domain generalization capabilities, even if the multi-dataset assessment validates the consistency of the suggested strategy across several benchmark datasets.

Comments-3: The reported performance metrics are promising, particularly on the DPFD-DET and ZJU-Leaper datasets, where the model achieved mAP@0.5 values of 93.6% and 93.0%, respectively. However, additional statistical validation, such as cross-validation or significance testing, would strengthen confidence in the results.

Reply-3: We accept the reviewer's recommendation for more statistical confirmation. An ablation study can not replace statistical validation methods like k-fold cross-validation or significance testing, even though it might shed light on the role of certain training methodologies and hyperparameter values. Extensive statistical validation was not carried out because the main goal of this study was to assess the efficacy of the suggested framework on various textile defect datasets; this is acknowledged as a shortcoming. Cross-validation and statistical significance analyses will be used in future research to evaluate the current results' robustness and repeatability.

Comments-4: The manuscript would benefit from a clearer description of the architectural novelty of UniDefectNet-Omni. Although the study presents YOLOv12-based defect detection, the distinction between the proposed framework and the baseline YOLOv12 architecture is not sufficiently highlighted.

Reply-4: We appreciate the reviewer's comment. The suggested system, which is based on the conventional YOLOv12 detector, aims to increase the robustness of defect identification by using adaptive augmentation, high-resolution feature learning, improved training techniques, and heterogeneous textile fault representation. The updated approach makes it clear that UniDefectNet-Omni's value is not in architectural redesign but rather in its unified learning framework and defect generalization capacity. The paper has been revised to provide a clear distinction between the suggested training and assessment framework and the baseline YOLOv12 architecture.

Comments-5: The paper contains numerous grammatical, formatting, and language issues that affect readability and scientific presentation. A thorough English language revision and careful proofreading are recommended before publication. For example,

several sections contain repetitive statements, inconsistent terminology, and awkward sentence constructions.

Reply-5: We sincerely thank the reviewer for this valuable and constructive feedback. The complete manuscript has been carefully revised to improve language quality, readability, and scientific presentation. Grammatical errors, repetitive statements, inconsistent terminology, and awkward sentence constructions have been thoroughly corrected. In addition, the manuscript has been carefully proofread to ensure clarity, consistency, and improved overall flow throughout the document.
