# Supplementary material for: Fabric defect detection using fine-tuned Yolo-12
Source: PLoS One. 2026 Jul 22;21(7):e0353550. doi: 10.1371/journal.pone.0353550 (PMC13390829; doi:10.1371/journal.pone.0353550)
Supplement: Attachment — Submitted filename: rs (1).docx [file pone.0353550.s001.docx]

Fabric Defect Detection using Fine-Tuned Yolo-12

1. The manuscript addresses a highly relevant industrial problem by proposing a unified defect detection framework (UniDefectNet-Omni) capable of identifying defects in both plain and printed fabrics, which is a notable contribution given that most previous studies focus on only one fabric category.
2. The evaluation on multiple datasets (Chenab Textile, TILDA v2, DPFD-DET, and ZJU-Leaper) demonstrates the model's generalization capability and provides comprehensive validation across diverse textile defect types.
3. The reported performance metrics are promising, particularly on the DPFD-DET and ZJU-Leaper datasets, where the model achieved mAP@0.5 values of 93.6% and 93.0%, respectively. However, additional statistical validation, such as cross-validation or significance testing, would strengthen confidence in the results.
4. The manuscript would benefit from a clearer description of the architectural novelty of UniDefectNet-Omni. Although the study presents YOLOv12-based defect detection, the distinction between the proposed framework and the baseline YOLOv12 architecture is not sufficiently highlighted.
5. The paper contains numerous grammatical, formatting, and language issues that affect readability and scientific presentation. A thorough English language revision and careful proofreading are recommended before publication. For example, several sections contain repetitive statements, inconsistent terminology, and awkward sentence constructions.
