# Supplementary material for: Fabric defect detection using fine-tuned Yolo-12
Source: PLoS One. 2026 Jul 22;21(7):e0353550. doi: 10.1371/journal.pone.0353550 (PMC13390829; doi:10.1371/journal.pone.0353550)
Supplement: Peer review history

# Peer review: Decision Letter 0

**DOI:** 10.1371/journal.pone.0353550.r001

**Authors:** Gaurav Arora

4 Jun 2026

PONE-D-26-20542

Fabric Defect Detection using Fine-Tuned Yolo-12

PLOS One

Dear Dr. Ashraf,

Thank you for submitting your manuscript to PLOS ONE. After careful consideration, we feel that it has merit but does not fully meet PLOS ONE’s publication criteria as it currently stands. Therefore, we invite you to submit a revised version of the manuscript that addresses the points raised during the review process.

==============================

**ACADEMIC EDITOR:**

Dear Authors,

The manuscript has been accessed, and major revision has been suggested by the respected reviewers.

Kindly address the comments and submit the revision timely.

==============================

Please submit your revised manuscript by Aug 03 2026 11:59PM. If you will need more time than this to complete your revisions, please reply to this message or contact the journal office at plosone@plos.org. When you're ready to submit your revision, log on to https://www.editorialmanager.com/pone/ and select the 'Submissions Needing Revision' folder to locate your manuscript file.

Please include the following items when submitting your revised manuscript:

- A letter that responds to each point raised by the academic editor and reviewer(s). You should upload this letter as a separate file labeled 'Response to Reviewers'.
- A marked-up copy of your manuscript that highlights changes made to the original version. You should upload this as a separate file labeled 'Revised Manuscript with Track Changes'.
- An unmarked version of your revised paper without tracked changes. You should upload this as a separate file labeled 'Manuscript'.

If you would like to make changes to your financial disclosure, please include your updated statement in your cover letter. Guidelines for resubmitting your figure files are available below the reviewer comments at the end of this letter.

If applicable, we recommend that you deposit your laboratory protocols in protocols.io to enhance the reproducibility of your results. Protocols.io assigns your protocol its own identifier (DOI) so that it can be cited independently in the future. For instructions see: https://journals.plos.org/plosone/s/submission-guidelines#loc-laboratory-protocols. Additionally, PLOS ONE offers an option for publishing peer-reviewed Lab Protocol articles, which describe protocols hosted on protocols.io. Read more information on sharing protocols at https://plos.org/protocols?utm_medium=editorial-email&utm_source=authorletters&utm_campaign=protocols.

As the corresponding author, your ORCID iD is verified in the submission system and will appear in the published article. PLOS supports the use of ORCID, and we encourage all coauthors to register for an ORCID iD and use it as well. Please encourage your coauthors to verify their ORCID iD within the submission system before final acceptance, as unverified ORCID iDs will not appear in the published article. *Only*  the individual author can complete the verification step; PLOS staff *cannot*  verify ORCID iDs on behalf of authors.

We look forward to receiving your revised manuscript.

Kind regards,

Gaurav Arora, Ph.D

Academic Editor

PLOS One

Journal Requirements:

When submitting your revision, we need you to address these additional requirements.

1. Please ensure that your manuscript meets PLOS ONE's style requirements, including those for file naming. The PLOS ONE style templates can be found at

https://journals.plos.org/plosone/s/file?id=wjVg/PLOSOne_formatting_sample_main_body.pdf and

https://journals.plos.org/plosone/s/file?id=ba62/PLOSOne_formatting_sample_title_authors_affiliations.pdf

2. Please note that PLOS One has specific guidelines on code sharing for submissions in which author-generated code underpins the findings in the manuscript. In these cases, we expect all author-generated code to be made available without restrictions upon publication of the work. Please review our guidelines at https://journals.plos.org/plosone/s/materials-and-software-sharing#loc-sharing-code and ensure that your code is shared in a way that follows best practice and facilitates reproducibility and reuse.

3. We notice that your figures are uploaded with the file type supplementary figures '. Please amend the file type to ‘Figure’.

4. In this instance it seems there may be acceptable restrictions in place that prevent the public sharing of your minimal data. However, in line with our goal of ensuring long-term data availability to all interested researchers, PLOS’ Data Policy states that authors cannot be the sole named individuals responsible for ensuring data access (http://journals.plos.org/plosone/s/data-availability#loc-acceptable-data-sharing-methods)

Data requests to a non-author institutional point of contact, such as a data access or ethics committee, helps guarantee long term stability and availability of data. Providing interested researchers with a durable point of contact ensures data will be accessible even if an author changes email addresses, institutions, or becomes unavailable to answer requests.

Before we proceed with your manuscript, please also provide non-author contact information (phone/email/hyperlink) for a data access committee, ethics committee, or other institutional body to which data requests may be sent. If no institutional body is available to respond to requests for your minimal data, please consider if there any institutional representatives who did not collaborate in the study, and are not listed as authors on the manuscript, who would be able to hold the data and respond to external requests for data access? If so, please provide their contact information (i.e., email address). Please also provide details on how you will ensure persistent or long-term data storage and availability.

5. If the reviewer comments include a recommendation to cite specific previously published works, please review and evaluate these publications to determine whether they are relevant and should be cited. There is no requirement to cite these works unless the editor has indicated otherwise.

Additional Editor Comments :

Dear Authors,

The manuscript has been accessed, and major revision has been suggested by the respected reviewers.

Kindly address the comments and submit the revision timely.

[Note: HTML markup is below. Please do not edit.]

Reviewers' comments:

Reviewer's Responses to Questions

**Comments to the Author**

1. Is the manuscript technically sound, and do the data support the conclusions?

The manuscript must describe a technically sound piece of scientific research with data that supports the conclusions. Experiments must have been conducted rigorously, with appropriate controls, replication, and sample sizes. The conclusions must be drawn appropriately based on the data presented.

Reviewer #1: Yes

Reviewer #2: Yes

**********

2. Has the statistical analysis been performed appropriately and rigorously?

Reviewer #1: I Don't Know

Reviewer #2: Yes

**********

3. Have the authors made all data underlying the findings in their manuscript fully available?

The PLOS Data policy requires authors to make all data underlying the findings described in their manuscript fully available without restriction, with rare exception (please refer to the Data Availability Statement in the manuscript PDF file). The data should be provided as part of the manuscript or its supporting information, or deposited to a public repository. For example, in addition to summary statistics, the data points behind means, medians and variance measures should be available. If there are restrictions on publicly sharing data—e.g. participant privacy or use of data from a third party—those must be specified.

Reviewer #1: Yes

Reviewer #2: Yes

**********

4. Is the manuscript presented in an intelligible fashion and written in standard English?

PLOS ONE does not copyedit accepted manuscripts, so the language in submitted articles must be clear, correct, and unambiguous. Any typographical or grammatical errors should be corrected at revision, so please note any specific errors here.

Reviewer #1: Yes

Reviewer #2: Yes

**********

5. Review Comments to the Author

Please use the space provided to explain your answers to the questions above. You may also include additional comments for the author, including concerns about dual publication, research ethics, or publication ethics. (Please upload your review as an attachment if it exceeds 20,000 characters)

Reviewer #1: 1. The novelty of UniDefectNet-Omni should be more clearly articulated, particularly in comparison with existing YOLO-based and transformer-based defect detection models.

2. The title is appropriate, but the manuscript inconsistently refers to the model as UniDefectNet-Omni, YOLOv12, and Yolo-12, which should be standardized throughout.

3. The abstract should explicitly describe the architectural modifications introduced beyond the baseline YOLOv12 model.

4. The literature review is extensive, but it is overly verbose and contains numerous grammatical errors that obscure the main research gap.

5. Author should add more about machine learning in literature review section. You may refer “Review of machine learning approaches for predicting mechanical behavior of composite materials”, Investigation of Tribological Behaviour of B4C–Graphene Glass Fiber Composites Using Taguchi, “Multi-Criteria Decision Making, and Machine Learning Approaches”, “Predictive modeling of thermoplastic nanocomposites using machine learning algorithms”.

6. Table 1 is useful, but several performance metrics are reported inconsistently and should be verified against the cited references.

7. The methodology does not clearly explain what architectural changes distinguish UniDefectNet-Omni from the standard YOLOv12 implementation.

8. The removal of rare defect classes may limit the generalization capability of the proposed model and should be discussed as a limitation.

9. The statement that additional public images were added after observing poor class performance introduces potential data leakage and should be carefully clarified.

10. Hyperparameter selection appears empirical, and no ablation study is provided to justify the chosen settings.

11. Several equations in the methodology section are standard definitions and do not add substantial value; instead, more implementation details should be provided.

12. Equation 8 contains a notation inconsistency where the same variable is used on both sides of the equation.

13. The manuscript should specify the computational resources used for training, including GPU model, memory, and total training time.

14. Results are presented only for the proposed model without direct benchmarking against baseline models such as YOLOv8, YOLOv11, or Faster R-CNN under identical conditions.

15. The claim that the model is “state-of-the-art” is not justified solely by showing one prediction example with high confidence.

16. Confidence scores of individual detections do not constitute evidence of superior model performance.

17. The comparison with prior studies should use consistent datasets and evaluation metrics to ensure fairness.

18. The ZJU-Leaper dataset table is incorrectly labeled as “DPFD-DET dataset” and should be corrected.

19. The manuscript reports exceptionally high mAP values for several classes (0.995), which may indicate overfitting and should be discussed.

20. The confusion matrix analysis is descriptive but does not provide deeper insight into the causes of misclassification.

21. No inference speed (FPS) or latency results are provided, despite claims of suitability for real-time industrial deployment.

22. Cross-dataset generalization is claimed, but no transfer learning or domain adaptation experiments are presented.

23. The practical significance of the proposed approach for textile manufacturers should be discussed in terms of deployment feasibility and economic impact.

24. Figures and tables should be carefully proofread for labeling inconsistencies and formatting issues.

25. The manuscript contains extensive grammatical and typographical errors and requires substantial English language editing.

26. Overall, the study is promising and industrially relevant, but major revisions are required to clarify the methodological novelty, strengthen comparative evaluation, and improve the presentation quality before publication.

Reviewer #2: 1. The manuscript addresses a highly relevant industrial problem by proposing a unified defect detection framework (UniDefectNet-Omni) capable of identifying defects in both plain and printed fabrics, which is a notable contribution given that most previous studies focus on only one fabric category.

2. The evaluation on multiple datasets (Chenab Textile, TILDA v2, DPFD-DET, and ZJU-Leaper) demonstrates the model's generalization capability and provides comprehensive validation across diverse textile defect types.

3. The reported performance metrics are promising, particularly on the DPFD-DET and ZJU-Leaper datasets, where the model achieved mAP@0.5 values of 93.6% and 93.0%, respectively. However, additional statistical validation, such as cross-validation or significance testing, would strengthen confidence in the results.

4. The manuscript would benefit from a clearer description of the architectural novelty of UniDefectNet-Omni. Although the study presents YOLOv12-based defect detection, the distinction between the proposed framework and the baseline YOLOv12 architecture is not sufficiently highlighted.

5. The paper contains numerous grammatical, formatting, and language issues that affect readability and scientific presentation. A thorough English language revision and careful proofreading are recommended before publication. For example, several sections contain repetitive statements, inconsistent terminology, and awkward sentence constructions.

**********

6. PLOS authors have the option to publish the peer review history of their article (what does this mean?). If published, this will include your full peer review and any attached files.

If you choose “no”, your identity will remain anonymous but your review may still be made public.

**Do you want your identity to be public for this peer review?**  For information about this choice, including consent withdrawal, please see our Privacy Policy.

Reviewer #1: No

Reviewer #2: **Yes:** Ravi Sevak

**********

[NOTE: If reviewer comments were submitted as an attachment file, they will be attached to this email and accessible via the submission site. Please log into your account, locate the manuscript record, and check for the action link "View Attachments". If this link does not appear, there are no attachment files.]

To ensure your figures meet our technical requirements, please review our figure guidelines: https://journals.plos.org/plosone/s/figures

You may also use PLOS’s free figure tool, NAAS, to help you prepare publication quality figures: https://journals.plos.org/plosone/s/figures#loc-tools-for-figure-preparation.

NAAS will assess whether your figures meet our technical requirements by comparing each figure against our figure specifications.

Attachment Submitted filename: rs (1).docx

# Author comment: Author response to Decision Letter 1

**DOI:** 10.1371/journal.pone.0353550.r002

19 Jun 2026

Response to Reviewers

Reviewer #1:

Comments-1:The novelty of UniDefectNet-Omni should be more clearly articulated, particularly in comparison with existing YOLO-based and transformer-based defect detection models.

Reply-1:.Thanks for the reviewer valuable suggestion. In response, we have further clarified novelty of UniDefectNet-Omni's distinctive defect representation architecture enables a single YOLOv12-based model to accurately detect a broad spectrum of textile faults across different fabric qualities, including plain, printed, multicolored, and grayscale textiles. UniDefectNet-Omni demonstrates dependable and consistent performance across a range of heterogeneous textile datasets while maintaining the efficiency required for industrial deployment, in contrast to transformer-based approaches that rely on computationally demanding attention mechanisms and existing YOLO-based models that are usually tested on a single fabric domain. Furthermore, compared with transformer-based defect detection models that rely on computationally intensive attention mechanisms and require higher computational resources, UniDefectNet-Omni maintains a favorable trade-off between detection accuracy, robustness, and computational efficiency, making it suitable for real-time industrial textile inspection The novelty has been included in the comparison on page # 44.

Comments-2: The title is appropriate, but the manuscript inconsistently refers to the model as UniDefectNet-Omni, YOLOv12, and Yolo-12, which should be standardized throughout.

Reply-2:.We thank the reviewer for highlighting this inconsistency. The proposed framework has been consistently referred to as UniDefectNet-Omni throughout the revised manuscript, while YOLOv12 has been used exclusively to denote the underlying backbone architecture. All inconsistent references, including variations such as "YOLO-12" and other naming discrepancies, have been carefully corrected to ensure uniform terminology and improve the overall clarity and consistency of the manuscript.

Comments-3: The abstract should explicitly describe the architectural modifications introduced beyond the baseline YOLOv12 model.

Reply-3:.We thank the reviewer for this important observation. The abstract has been revised to more clearly articulate the nature of the proposed improvements with respect to the baseline YOLOv12 framework. The abstract has been revised to clarify that the proposed approach, which builds on the conventional YOLOv12 architecture, enhances fault representation through dataset variety, high-resolution learning, and improved training procedures rather than architectural change.

Comments-4: The literature review is extensive, but it is overly verbose and contains numerous grammatical errors that obscure the main research gap.

Reply-4:.We sincerely thank the reviewer for this valuable suggestion. The literature review section has been thoroughly revised to improve clarity, conciseness, and grammatical accuracy. Redundant and overly verbose content has been removed, and the discussion has been streamlined to better highlight the key research gap and positioning of the proposed work within the existing literature. The revised section now presents a more focused and coherent narrative aligned with the objectives of the study.

Comments-5: Author should add more about machine learning in literature review section. You may refer “Review of machine learning approaches for predicting mechanical behavior of composite materials”, Investigation of Tribological Behaviour of B4C–Graphene Glass Fiber Composites Using Taguchi, “Multi-Criteria Decision Making, and Machine Learning Approaches”, “Predictive modeling of thermoplastic nanocomposites using machine learning algorithms”.

Reply-5:.The relevant studies, “Review of machine learning approaches for predicting mechanical behavior of composite materials” and Predictive modeling of thermoplastic nanocomposites using machine learning algorithms have been included.

Comments-6: Table 1 is useful, but several performance metrics are reported inconsistent and should be verified against the cited references.

Reply-6:We thank the reviewer for this careful observation. Table 1 has been thoroughly reviewed and cross-checked against the original cited references to ensure accuracy and consistency of all reported performance metrics. The table has been improved and corrected.

Comments-7: The methodology does not clearly explain what architectural changes distinguish UniDefectNet-Omni from the standard YOLOv12 implementation.

Reply-7: We appreciate the reviewer's comment. The suggested system, which is based on the conventional YOLOv12 detector, aims to increase the robustness of defect identification by using adaptive augmentation, high-resolution feature learning, improved training techniques, and heterogeneous textile fault representation. The updated approach makes it clear that UniDefectNet-Omni's value is not in architectural redesign but rather in its unified learning framework and defect generalization capacity. The paper has been revised to provide a clear distinction between the suggested training and assessment framework and the baseline YOLOv12 architecture.

Comments-8: The removal of rare defect classes may limit the generalization capability of the proposed model and should be discussed as a limitation.

Reply-8: We appreciate the reviewer's insightful comment. The article now includes a restriction paragraph that acknowledges that the model's capacity to generalize to occasional or undetectable faults in actual textile production may be diminished by the absence of exceedingly rare defect types.

Comments-9: The statement that additional public images were added after observing poor class performance introduces potential data leakage and should be carefully clarified.

Reply-9: We thank the reviewer for this important concern. We would like to clarify that no additional images were introduced into the dataset during the experiments in a manner that could lead to data leakage or bias. The statement in the manuscript refers solely to the description of the original dataset construction process as reported in prior work. It was included for completeness and context, not as part of any modification to the dataset used in our experiments. All experiments in this study were conducted using the publicly available dataset as-is, without any alteration, augmentation of external images, or class-balancing through additional data collection. We have revised the manuscript to clarify this point more explicitly to avoid any misunderstanding regarding dataset integrity and experimental validity. This statement is refered to original dataset that how dataset built. We just describe dataset creation as reference. We did not add anything to dataset.

Comments-10: Hyperparameter selection appears empirical, and no ablation study is provided to justify the chosen settings.

Reply-10: We value the reviewer's insightful comment. The selected hyperparameters were first determined using a variety of exploratory tests and recommendations from the YOLOv12 training framework. We acknowledge that the text did a poor job of explaining the logic behind these choices. To alleviate this concern, an ablation study has been incorporated to evaluate the impact of significant hyperparameters, including learning rate, batch size, picture quality, data augmentation intensity, and warm-up epochs. The results demonstrate that the selected design provides the greatest feasible balance between convergence stability, detection accuracy, and processing efficiency. The ablation analysis and associated discussion are now included in the revised text.

Comments-11: Several equations in the methodology section are standard definitions and do not add substantial value; instead, more implementation details should be provided.

Reply-11: We value the reviewer's wise suggestion. We have revised the method section by removing unnecessary textbook-level equations to highlight implementation-specific designs, including the true training scheduling configuration, optimizer actions, and parameter settings used in this YOLOv12-based approach. Additional details with learning rate

schedules, momentum configuration, and execution-level learning technique have been incorporated to improve repeatability and simplicity.

Comments-12: Equation 8 contains a notation inconsistency where the same variable is used on both sides of the equation.

Reply-12: We sincerely appreciate the reviewer's perceptive evaluation. We agree that the prior version of Equation 8, now Equation 9, created a notation inconsistency that might lead to misconceptions by using the symbol "wulr" as both the function output and part of the variable name. To overcome this challenge, we have revised the equation using a commonly used and comprehensible mathematical language. The warm-up learning rate is now described as a function of the epoch, and the warm-up length is represented by a separate parameter.

Comments-13: The manuscript should specify the computational resources used for training, including GPU model, memory, and total training time.

Reply-13: We are grateful for the reviewer's important recommendation. We have also given a detailed description of the computational architecture used for model training in order to improve experimental reproducibility and transparency. The Kaggle platform's default GPU setup was used for each experiment. Specifically, the model was trained on a Kaggle GPU T4 accelerator with about 16 GB of GPU RAM. For the training process, Kaggle laptops were utilized rather than an external high-performance computer cluster. The total training time for the proposed model, including the stages of data loading, augmentation, and assessment, was about 8 hours.

Comments-14: Results are presented only for the proposed model without direct benchmarking against baseline models such as YOLOv8, YOLOv11, or Faster R-CNN under identical conditions.

Reply-14: The proposed model is trained on the Chenab textile dataset and validated with three datasets, such as TildaV2, DPFD-DET, and ZJU-Leaper. The model is comprehensively validated.

Comments-15: Furthermore, a detailed comparative analysis is performed in Table 14.The claim that the model is “state-of-the-art” is not justified solely by showing one prediction example with high confidence.

Reply-15: The proposed solution has the ability of a unified strategy for detection by combining high-resolution training, diverse representations, and adaptive augmentation to enhance the performance of detection across various fabric defect categories, characteristics, and generalization capacity. According to a comprehensive review of literature, and to the best of our knowledge, there is no such model that addresses such defect detection through a single model.

Comments-16: Confidence scores of individual detections do not constitute evidence of superior model performance.

Reply-16: We appreciate the reviewer's insightful comments. We concur that better model performance cannot be inferred only from the confidence scores of individual detection cases. The qualitative detection numbers provided a visual representation of the model's capacity to locate and identify textile flaws under typical circumstances. We use quantitative indicators, including accuracy, recall, F1-score, mAP@0.5, mAP@0.5:0.95, confusion matrix analysis, and loss convergence behavior to assess performance objectively. We have explicitly stated in the updated publication that the confidence levels displayed in qualitative examples are just meant for visualization and should not be taken as proof of the overall superiority of the model. To prevent such misunderstandings, corresponding explanation materials have been provided.

Comments-17: The comparison with prior studies should use consistent datasets and evaluation metrics to ensure fairness.

Reply-17: We sincerely thank the reviewer for this important suggestion. The comparative study has been carefully revised to improve fairness, consistency, and methodological rigor. We have ensured that all reported comparisons are aligned in terms of datasets and evaluation metrics, or clearly annotated where differences exist due to variations in original study settings. In addition, the revised manuscript explicitly standardizes the evaluation criteria used for benchmarking, enabling a more reliable and transparent comparison with prior work. These improvements strengthen the validity and interpretability of the reported results. The comparative study has been updated and improved.

Comments-18: The ZJU-Leaper dataset table is incorrectly labeled as “DPFD-DET dataset” and should be corrected.

Reply-18: We thank the reviewer for carefully reviewing the manuscript.In revised manuscript, we have coreected and updated as per your suggestion.

Comments-19: The manuscript reports exceptionally high mAP values for several classes (0.995), which may indicate overfitting and should be discussed.

Reply-19: We value the reviewer's informative feedback. We acknowledge that concerns regarding potential overfitting may arise from abnormally high mAP values for some fault classes. However, because these classes correspond to flaws with well-recognizable visual characteristics and sufficient training data, they are rather simple to identify. To decrease overfitting during training, regularization techniques such as weight decay, dropout, data augmentation, and early stopping were employed. We do agree, though, that exceptionally high class-wise mAP values may partially reflect the dataset

Comments-20: The confusion matrix analysis is descriptive but does not provide deeper insight into the causes of misclassification.

Reply-20: We appreciate the reviewer's insightful comments. We recognize that excessively high mAP values for some fault classes may raise questions about possible overfitting. However, these classes are rather easy to detect as they correlate to defects with easily identifiable visual traits and enough training data. Regularization strategies such as weight decay, dropout, data augmentation, and early halting were used to reduce overfitting during training. However, we acknowledge that extraordinarily high class-wise mAP values might be a partial reflection of the dataset.

Comments-21: No inference speed (FPS) or latency results are provided, despite claims of suitability for real-time industrial deployment.

Reply-21: We appreciate the reviewer bringing this significant problem to our attention. We concur that when assessing whether a defect detection approach is appropriate for real-time industrial deployment, inference speed and latency are crucial factors. The original publication did not provide thorough runtime benchmarking because the present study's primary focus is on detection accuracy and resilience across varied textile fault datasets.

Comments-22: Cross-dataset generalization is claimed, but no transfer learning or domain adaptation experiments are presented.

Reply-22: We appreciate the reviewer's insightful comment. We concur that transfer learning, domain adaptation, or cross-domain assessment experiments in which a model trained on one dataset is assessed on another dataset without retraining are usually necessary for thorough validation of cross-dataset generalization. The phrase "cross-dataset" was used in the current study to denote that the suggested framework was assessed on many textile defect datasets with various imaging circumstances and defect characteristics.

Comments-23: The practical significance of the proposed approach for textile manufacturers should be discussed in terms of deployment feasibility and economic impact.

Reply-23: In order to emphasize the usefulness of the suggested framework for textile manufacturing settings, we have extended the conclusion in the updated manuscript. In particular, we address how automated defect identification may eliminate material loss related to faulty fabric rolls, enhance product quality consistency, enable early detection of production problems, and lessen the need for manual inspection. We value the reviewer's wise suggestio

Attachment Submitted filename: Response to Reviewers.pdf

# Peer review: Decision Letter 1

**DOI:** 10.1371/journal.pone.0353550.r003

**Authors:** Gaurav Arora

25 Jun 2026

Fabric Defect Detection using Fine-Tuned Yolo-12

PONE-D-26-20542R1

Dear Dr. Ashraf,

We’re pleased to inform you that your manuscript has been judged scientifically suitable for publication and will be formally accepted for publication once it meets all outstanding technical requirements.

Within one week, you’ll receive an e-mail detailing the required amendments. When these have been addressed, you’ll receive a formal acceptance letter and your manuscript will be scheduled for publication.

An invoice will be generated when your article is formally accepted. Please note, if your institution has a publishing partnership with PLOS and your article meets the relevant criteria, all or part of your publication costs will be covered. Please make sure your user information is up-to-date by logging into Editorial Manager at Editorial Manager® and clicking the ‘Update My Information' link at the top of the page. For questions related to billing, please contact billing support.

If your institution or institutions have a press office, please notify them about your upcoming paper to help maximize its impact. If they’ll be preparing press materials, please inform our press team as soon as possible -- no later than 48 hours after receiving the formal acceptance. Your manuscript will remain under strict press embargo until 2 pm Eastern Time on the date of publication. For more information, please contact onepress@plos.org.

Kind regards,

Gaurav Arora, Ph.D

Academic Editor

PLOS One

Additional Editor Comments (optional):

The reviewers have given the acceptance for the article.

Reviewers' comments:

Reviewer's Responses to Questions

**Comments to the Author**

1. If the authors have adequately addressed your comments raised in a previous round of review and you feel that this manuscript is now acceptable for publication, you may indicate that here to bypass the “Comments to the Author” section, enter your conflict of interest statement in the “Confidential to Editor” section, and submit your "Accept" recommendation.

Reviewer #1: All comments have been addressed

Reviewer #2: All comments have been addressed

**********

2. Is the manuscript technically sound, and do the data support the conclusions?

The manuscript must describe a technically sound piece of scientific research with data that supports the conclusions. Experiments must have been conducted rigorously, with appropriate controls, replication, and sample sizes. The conclusions must be drawn appropriately based on the data presented.

Reviewer #1: Yes

Reviewer #2: Yes

**********

3. Has the statistical analysis been performed appropriately and rigorously?

Reviewer #1: N/A

Reviewer #2: Yes

**********

4. Have the authors made all data underlying the findings in their manuscript fully available?

The PLOS Data policy requires authors to make all data underlying the findings described in their manuscript fully available without restriction, with rare exception (please refer to the Data Availability Statement in the manuscript PDF file). The data should be provided as part of the manuscript or its supporting information, or deposited to a public repository. For example, in addition to summary statistics, the data points behind means, medians and variance measures should be available. If there are restrictions on publicly sharing data—e.g. participant privacy or use of data from a third party—those must be specified.

Reviewer #1: Yes

Reviewer #2: Yes

**********

5. Is the manuscript presented in an intelligible fashion and written in standard English?

PLOS ONE does not copyedit accepted manuscripts, so the language in submitted articles must be clear, correct, and unambiguous. Any typographical or grammatical errors should be corrected at revision, so please note any specific errors here.

Reviewer #1: Yes

Reviewer #2: Yes

**********

6. Review Comments to the Author

Please use the space provided to explain your answers to the questions above. You may also include additional comments for the author, including concerns about dual publication, research ethics, or publication ethics. (Please upload your review as an attachment if it exceeds 20,000 characters)

Reviewer #1: The authors have addressed all the comments carefully. Now the manuscript can be accpeted for the publication.

Reviewer #2: (No Response)

**********

7. PLOS authors have the option to publish the peer review history of their article (what does this mean?). If published, this will include your full peer review and any attached files.

If you choose “no”, your identity will remain anonymous but your review may still be made public.

**Do you want your identity to be public for this peer review?**  For information about this choice, including consent withdrawal, please see our Privacy Policy.

Reviewer #1: No

Reviewer #2: No

**********

# Editor report: Acceptance letter

**DOI:** 10.1371/journal.pone.0353550.r004

**Authors:** Gaurav Arora

PONE-D-26-20542R1

PLOS One

Dear Dr. Ashraf,

I'm pleased to inform you that your manuscript has been deemed suitable for publication in PLOS One. Congratulations! Your manuscript is now being handed over to our production team.

At this stage, our production department will prepare your paper for publication. This includes ensuring the following:

* All references, tables, and figures are properly cited

* All relevant supporting information is included in the manuscript submission,

* There are no issues that prevent the paper from being properly typeset

You will receive further instructions from the production team, including instructions on how to review your proof when it is ready. Please keep in mind that we are working through a large volume of accepted articles, so please give us a few days to review your paper and let you know the next and final steps.

Lastly, if your institution or institutions have a press office, please let them know about your upcoming paper now to help maximize its impact. If they'll be preparing press materials, please inform our press team within the next 48 hours. Your manuscript will remain under strict press embargo until 2 pm Eastern Time on the date of publication. For more information, please contact onepress@plos.org.

You will receive an invoice from PLOS for your publication fee after your manuscript has reached the completed accept phase. If you receive an email requesting payment before acceptance or for any other service, this may be a phishing scheme. Learn how to identify phishing emails and protect your accounts at https://explore.plos.org/phishing.

If we can help with anything else, please email us at customercare@plos.org.

Thank you for submitting your work to PLOS ONE and supporting open access.

Kind regards,

PLOS ONE Editorial Office Staff

on behalf of

Dr. Gaurav Arora

Academic Editor

PLOS One
